# Defining the Age of Young Ischemic Stroke Using Data-Driven Approaches

**DOI:** 10.3390/jcm12072600

**Published:** 2023-03-30

**Authors:** Vida Abedi, Clare Lambert, Durgesh Chaudhary, Emily Rieder, Venkatesh Avula, Wenke Hwang, Jiang Li, Ramin Zand

**Affiliations:** 1 Department of Molecular and Functional Genomics, Weis Center for Research, Geisinger Health System, Danville, PA 17822, USA; 2 Department of Public Health Sciences, College of Medicine, The Pennsylvania State University, Hershey, PA 17033, USA; 3 Department of Neurology, Yale New Haven Hospital, New Haven, CT 06510, USA; 4 Geisinger Neuroscience Institute, Geisinger Health System, Danville, PA 17822, USA; 5 Department of Neurology, College of Medicine, The Pennsylvania State University, Hershey, PA 17033, USA; 6 Geisinger Commonwealth, School of Medicine, Scranton, PA 18509, USA

**Keywords:** ischemic stroke, data science, machine-learning, electronic health records, EHR

## Abstract

**Introduction**: The cut-point for defining the age of young ischemic stroke (IS) is clinically and epidemiologically important, yet it is arbitrary and differs across studies. In this study, we leveraged electronic health records (EHRs) and data science techniques to estimate an optimal cut-point for defining the age of young IS. **Methods:** Patient-level EHRs were extracted from 13 hospitals in Pennsylvania, and used in two parallel approaches. The first approach included ICD9/10, from IS patients to group comorbidities, and computed similarity scores between every patient pair. We determined the optimal age of young IS by analyzing the trend of patient similarity with respect to their clinical profile for different ages of index IS. The second approach used the IS cohort and control (without IS), and built three sets of machine-learning models—generalized linear regression (GLM), random forest (RF), and XGBoost (XGB)—to classify patients for seventeen age groups. After extracting feature importance from the models, we determined the optimal age of young IS by analyzing the pattern of comorbidity with respect to the age of index IS. Both approaches were completed separately for male and female patients. **Results:** The stroke cohort contained 7555 ISs, and the control included 31,067 patients. In the first approach, the optimal age of young stroke was 53.7 and 51.0 years in female and male patients, respectively. In the second approach, we created 102 models, based on three algorithms, 17 age brackets, and two sexes. The optimal age was 53 (GLM), 52 (RF), and 54 (XGB) for female, and 52 (GLM and RF) and 53 (RF) for male patients. Different age and sex groups exhibited different comorbidity patterns. **Discussion:** Using a data-driven approach, we determined the age of young stroke to be 54 years for women and 52 years for men in our mainly rural population, in central Pennsylvania. Future validation studies should include more diverse populations.

## 1. Introduction

From 1989 to 2008, there has been a 36–38% increase in “young strokes” in the United States [1]. However, the definition of “young stroke” differs from source to source, making it difficult to ascertain the incidence of ischemic stroke (IS) in young adults. Up to 10% of first-time ISs occur in people under the age of 45 [2], and nearly one-quarter of strokes occur in the working-age population [3]. Despite a fall in the overall prevalence of IS over the last decade, there has been a rise in “young strokes” [2]. More rigorous management of traditional IS risk factors, such as diabetes, hypertension, hypercholesterolemia, and smoking, may have contributed to a decline in whole-population stroke rates in older populations [4]; meanwhile, the prevalence of rarer etiologies that plague predominantly younger cohorts, such as hypercoagulable states, vasculitis, and genetic causes, Ref. [5] remain the same. Additionally, the prevalence of traditional risk factors may be shifting toward younger cohorts over time [6].

There is no standard definition of “young stroke”, and most studies have used an arbitrary upper cutoff between 45 and 59 years [7,8,9,10,11,12,13]. The age cutoff is clinically meaningful, since the uncommon stroke etiologies are more frequent among the younger population, and they may need a more comprehensive workup. The age cutoff is also epidemiologically meaningful to monitor the IS risk factors and incidence among different age brackets, and investigate rare causes, especially genetic predispositions. In our previous study on young stroke patients’ outcomes, we used multiple upper-age cut-points (43.7, 49.0, and 55.0 years) to define the young IS cohort [14]. The current study aimed to determine the age cut-point for defining “young stoke” in men and women, using a data-driven approach and comprehensive clinical data from a large, mainly rural, population in the United States. We further investigated the rare and traditional stroke risk factors among the young IS cohorts.

## 2. Methods

The study was conducted and reported according to the transparent reporting of a multivariable prediction model for individual prognosis or diagnosis (TRIPOD) guidelines [15]. TRIPOD provides a set of recommendations for the reporting of studies developing, validating, or updating a prediction model to improve transparency and reproducibility. The Supplemental Material (Table S1) includes the TRIPOD checklist. The study was reviewed and approved by the Geisinger Institutional Review Board to meet “non-human subject research” for using de-identified information.

We used two parallel approaches to determine and corroborate the age cut-point for young stroke. In the first approach, we used diagnosis codes based on ICD-9/10-CM (based on the entire comorbidity profile) from IS patients to group patient comorbidities, computed similarity scores between every patient pair, and determined the optimal age cut-point for defining young stroke by analyzing the trend of patient similarity with respect to their respective comorbidities for different age at IS. A similarity score was used to measure how every two patients were comparable based on their risk profiles. For the second approach, we used the IS patient cohort and controls (patients without IS) to build three sets of machine-learning (ML) models to classify patients with IS for 17 different age groups. We then extracted the feature importance from the models and determined the optimal age cut-point of young stroke by analyzing the pattern of comorbidity for different groups of patients based on the IS age of onset. The second strategy used clinical data based on common and rare vascular risk factors. Both approaches were performed separately for male and female patients. 

### 2.1. Data Source and Patient Population

Consecutive patient-level structured data from 13 hospitals of a large health system (Geisinger) in Pennsylvania, United States, from September 2003 to May 2019, were used for this study. Patients were included in the study if they were at least 18 years old at stroke onset and had no prior history of hemorrhagic or ischemic stroke. The patients were labeled as IS if they had an encounter at the Geisinger emergency department (ED) with a discharge diagnosis of ischemic stroke (IS), a brain MRI during their encounter, and an encounter duration of 24 h or more. The patients were labeled as control (without IS) if they had an ED encounter at Geisinger and an inpatient stay of at least 24 h with brain imaging (CT or MRI), but did not have a discharge diagnosis of stroke, transient ischemic attack (TIA), or intracerebral hemorrhage (ICH). For the second approach, different groups were created based on the patient’s age at the index encounter. All meta-data and code developed for this study are available in a GitHub repository: https://github.com/TheDecodeLab/YoungStroke (accessed on 20 March 2023).

### 2.2. Data Variables 

For the first approach, all the diagnosis codes, based on ICD-9-CM/ICD-10-CM, prior to index IS were extracted for all the IS patients; these codes were mapped to 534 Phecodes [16]. This data-driven strategy reduced the need to select associated factors manually. For the second approach, literature and experts were consulted, and a total of 23 variables (Table S2) were selected; this list was constructed according to the International Pediatric Stroke Study Classifications [17], and other sources [18,19,20,21,22].

The variables included the patient’s past medical history, demographics, and clinical data; all the variables were available and extracted (see Supplemental Material Table S3 for relevant ICD-9-CM/ICD-10-CM codes) from Geisinger’s electronic health record (EHR) data warehouse. Some variables were obtained only at the time of index stroke, others had a three-month buffer window after index stroke, and some were obtained if identified at any time in the patient’s medical record. The data variables and the extraction time frame are provided in Table S2. Variables that were given a three-month buffer window were those that could take time after hospital discharge to reach a definitive diagnosis, such as “hypercoagulable state”. Variables present at any time included those that afflicted patients lifelong, such as fibromuscular dysplasia. 

### 2.3. Data Pre-Processing and Imputation

Body Mass Index (BMI) information closest to the index ED encounter was dichotomized and used as a binary variable (normal: if BMI ≤ 25 or overweight: if BMI > 25). The BMI sustained 2.3% missingness, and an imputation was performed using the MICE (Multivariate Imputation by Chained Equations) package [23]. We have previously shown that, for missingness in the range in this study, MICE is an adequate choice for EHR [24,25]. 

### 2.4. Statistical Analysis 

All binary variables were presented as count and percentage. The prevalence of each variable within the cases was compared to controls, using a Pearson’s chi-squared test or Fisher’s exact test. A *p*-value of less than 0.05 was considered significant for all analyses. All statistical analyses were performed using R version 4.0.3 [26].

### 2.5. First Approach: Phenotype Modeling

As mentioned above, the ICD9-CM/ICD10-CM codes of patients with first-time IS were mapped to Phecode using Phecode Map 1.2 (R package ‘*PheWAS*’) [16]. After grouping patients by sex, a similarity score between every pair of patients was calculated using the matrix of Phecodes as the input, and cosine similarity as the scoring metric. The median similarity score for each patient was calculated and plotted versus the age of IS onset. The scatter plot was then analyzed to identify a trend of a similar pattern, using locally weighted regression (Loess regression) to fit a smoothed line. The estimated cut-point for approximating the age of young stroke was determined as the age best separating the patient similarity score by standardized Wilcoxon statistic, using the R package ‘*maxstat*’.

### 2.6. Second Approach: Machine Learning 

We used three algorithms—logistic regression (generalized linear models, GLM), in [27] extreme gradient boosting (XGB), [28] and random forest (RF) [29]—to train classifiers based on IS (cases) and non-IS patients (controls) for different age brackets. Following the classification, we extracted the feature importance, based on the 23 variables, for the different age groups; the models were created separately for males and females. The relative feature importance of each variable in each age bracket, and for males and females, was used to identify the changing comorbidity patterns. We created 102 models, based on 3 algorithms, 17 age brackets, and 2 sexes. The 17 age brackets included IS index age below 40, 45, 46, 47, 48, 49, 50, 51, 52, 53, 54, 55, 60, 65, 70, 75, and 80. Given that most studies used an age bracket in the range of 45–55, we designed our study to have a higher resolution for this age range. 

We split the data for each group into training (80%) and testing sets (20%). A parameter grid was built to train the model with five-fold repeated cross-validation, with five repeats. Model-tuning was performed by an automatic grid search, with five different values to try for each algorithm parameter randomly. Finally, 20% testing set data were used to calculate the model AUROC as a metric of model performance. The average feature importance was extracted from the fine-tuned models in each age and sex group. A heatmap was also generated to help visualize the patterns of change over time for the 23 variables.

A comparison of comorbidity patterns was performed by calculating the similarity measure between feature importance vectors for different age and sex groups. The similarity of the pattern of comorbidities among different groups can be a measure of how different these cohorts are, as the age of IS onset is varied from 40 to 80 years. The similarity was calculated using the cosine similarity measure. Finally, the non-parametric Wilcoxon statistic test was performed on the cosine similarity scores for different age brackets to determine the age cut-point, using the R package ‘*maxstat*’.

## 3. Results

### 3.1. Patient Population and Characteristics

The stroke cohort comprised 7555 IS patients who presented to Geisinger from 2003 to 2019, and met our inclusion criteria. The control cohort included 31,067 adult patients who presented to the Geisinger ED during the same period, and met the inclusion criteria for our control group. Females and males were analyzed separately. A total of 3892 males and 3663 females had IS during the study period; 66.3% of males and 50.1% of females were under the age of 75 at the first-time stroke, whereas 15.8% of males and 11.8% of females were under the age of 55 (Tables S4 and S5). 

The prevalence of certain risk factors did not differ significantly between cases (IS) compared to controls (without IS). For example, in the female cohorts, alcohol abuse or dependence, neoplasm, rheumatic disease, mood disorder, and giant cell arteritis (GCA) demonstrated no statistically significant difference in prevalence between cases and controls for any age brackets. In female patients, drug dependence or abuse was significantly different in the age brackets of <40 and <45, whereas current smoking was only different in the <80 and the all-ages brackets. For males, current smoking and GCA were the only variables that showed no statistical difference between cases and controls. 

**Figure 1:**
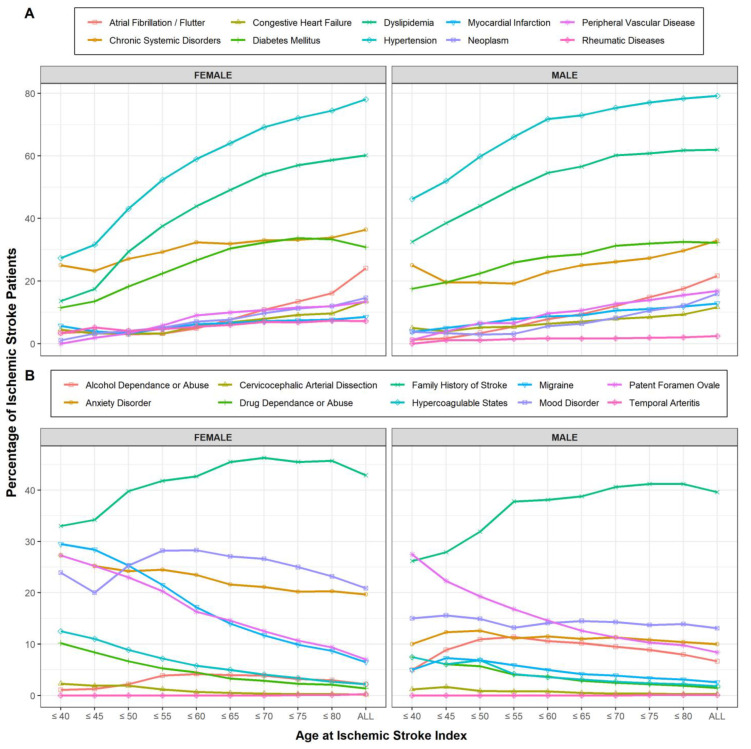
Line graph showing the prevalence of comorbidities as a percentage of the group total (*y*-axis) with respect to the age bracket increments (*x*-axis). (**A**) Line graph of first half of risk factors, (**B**) Lines graph of the second half of risk factors. All risk factors are spread over two graphs for clarity given high number of risk factors.

Generally, three main risk factors patterns were measured: (1) an increase in prevalence with increasing age, (2) a decrease in prevalence with increasing age, and (3) relative steadiness with increasing age. For example, in women, migraine (29.5% of <40 versus 9.7% of <80) and PFO (27.3% of <40 versus 9.4% of <80) demonstrated a declining prevalence. In men, PFO followed the same pattern (27.5% in <40 versus 9.8% in <80). In women, hypertension (7.3% in <40 versus 74.5% in <80), dyslipidemia (13.6% in <40 versus 58.7% in <80), and diabetes (11.4% in <40 versus 33.3% in <80) are examples of variables that demonstrated an opposite pattern, whereby the prevalence increased with age at IS onset. In men, hypertension (46.2% in <40 versus 78.9% in <80), dyslipidemia (32.5% in <40 versus 61.8% in <80), neoplasm (3.8% in <40 versus 12.1% in <80), and MI (3.8% in <40 versus 11.8% in <80) followed this pattern. Some variables, such as BMI, arteriopathies, cervicocephalic dissection in women, mood disorders, smoking, and cervicocephalic dissection in men, did not fluctuate with respect to IS age of onset. A list of variables and the respective percentages for the different age brackets in both males and females are summarized in Table S4 (females) and Table S5 (males). Figure 1A,B shows the changing prevalence of comorbidities with respect to the age of IS onset. 

### 3.2. Estimating the Age Cut-Point for Young IS Using Phenotype Modeling (Approach 1)

**Figure 2:**
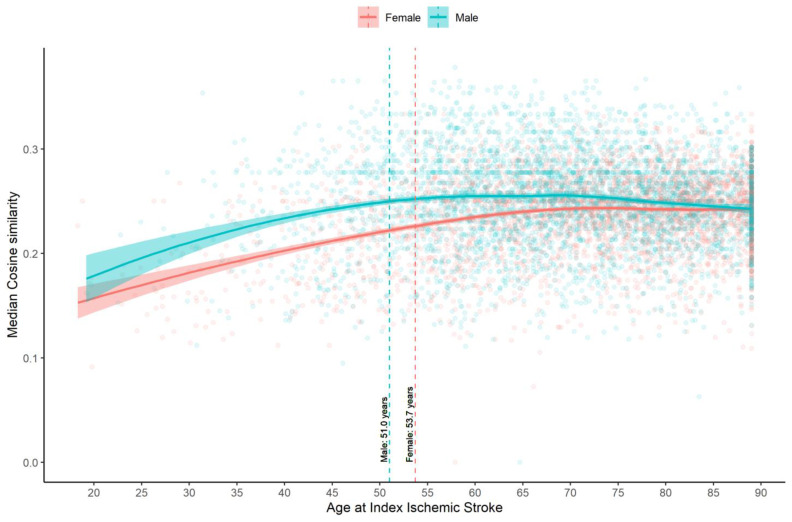
Scatter plot of median cosine similarity and age at index ischemic stroke date with a smoothed line, using loess regression.

The ICD-9-CM/ICD-10-CM codes of IS patients up to the index stroke date were mapped to 534 Phecodes. Apart from cerebrovascular disease, the top five Phecodes groups in the IS patients were hypertension (74%), disorders of lipid metabolism (61.9%), neurological disorders (38.1%), diabetes mellitus (31.6%), and cardiac dysrhythmias (30.4%). After calculating the cosine similarity based on the Phecodes variable, the age cut-point was estimated to be 53.7 years in female IS patients and 51.0 years in male IS patients (Figure 2).

### 3.3. Estimating the Age Cut-Point for Young IS Using Machine Learning (Approach 2)

Models can be developed to predict stroke using clinical data. In total, three algorithms (LR, XGB, and RF) were built for each age group (<40, <45, <46, <47, <48, <49, <50, <51, <52, <53, <54, <55, <60, <65, <70, <75, <80, and all), and for males and females separately, resulting in 102 models (three algorithms × 17 age brackets × two sexes). Supplemental Figure S1 summarizes the model AUROC and accuracy of each age/sex group in classifying stroke patients. Model parameters are summarized in Supplemental Material Table S6. In general, as the number of patients increased with the index age, the model AUROC increased steadily, reaching above 0.85. Model accuracy was less affected; however, the overall trend was slightly higher in models developed for females (overall accuracy above 0.8 was observed for all age brackets in the female group). Feature importance, extracted from these models for each age bracket and gender, was used to study the pattern of comorbidities, as the patients’ age at the index increased. 

**Figure 3:**
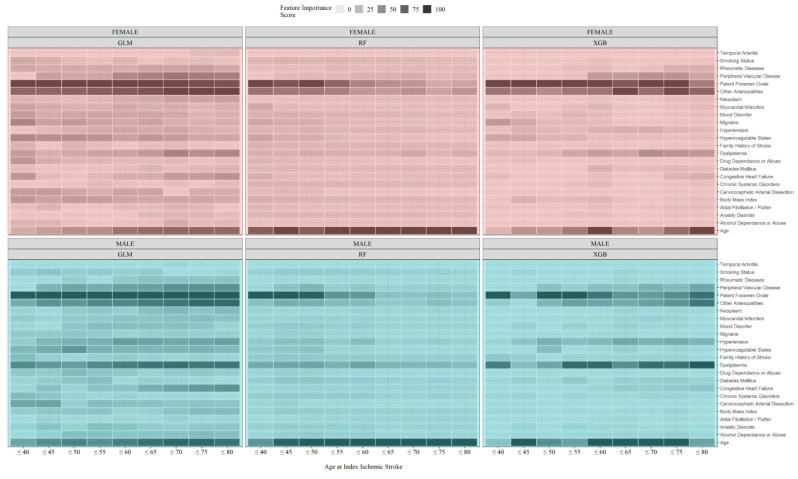
Average feature importance of the variables used by GLM, RF, and XGB models.

**Figure 4:**
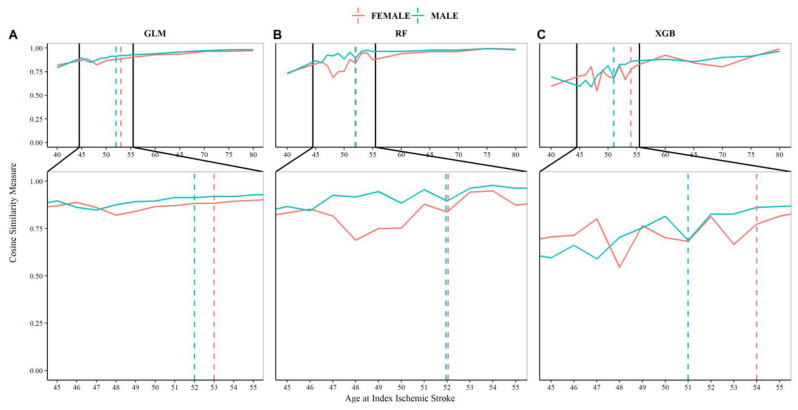
The trend of cosine similarity versus age at index ischemic stroke. Lower cosine similarity scores were indicative of increased dissimilarity, suggesting a change in risk factor profile across that age cutoff. (**A**) Cosine similarity trend for GLM model. (**B**) Cosine similarity trend for RF model. (**C**) Cosine similarity trend for XGB model.

Feature importance, extracted from ML-based models, can be used to estimate the age of “young stroke.” The pattern of feature importance can be visualized in a heatmap (Figure 3). To quantify the patterns observed in Figure 3, the similarity between the contribution of risk factors to the classification was measured for each age bracket using the cosine measure. More specifically, comparisons were made between the less-than age groups and the entire IS population. For instance, feature importance vectors for females <40 years were compared with females in all age groups. The resulting trends (trend of similarity measures for males and females), presented in Figure 4, showed the pattern of change in the associated factors based on the ML-based models. Our ML-based models showed that in females, the three models led to an age of 53 (based on GLM), 52 (based on RF), and 54 (based on XGB) for defining the age cutoff of young IS. In comparison, this pattern changed slightly for male patients: 52 years based on GLM and RF, and 53 based on the XGB model. These results corroborated our data-driven strategy based on phenotype modeling (approach 1) in defining the age of young stroke. 

## 4. Discussion

### 4.1. “Young Stroke” Cutoff

This is the first study using a data-driven strategy to identify the most appropriate age for young stroke in male and female patients, using a large population-based cohort. Two different approaches, based on phenotype analysis and supervised ML modeling, corroborated that young stroke has an optimal age cut-point, which is also slightly different in male and female patients. A more precise cut-point is 54 years for women and 52 years for men. This finding is important when analyzing clinical data or determining young stroke for epidemiological or genetic studies. Past literature has not defined an age cutoff that differentiates “young stroke” from the traditional stroke that occurs most often in elderly people, Ref. [30] and arbitrary age cutoffs have been applied across various studies [31]. A summary of important studies looking at young stroke, highlighting the variability of chosen age cutoffs, is included in Table S7. 

### 4.2. Vascular Risk Factors 

In our cohort, young patients had lower rates of traditional and higher rates of rare risk factors compared to older patients. For example, hypertension and dyslipidemia existed at a lower prevalence in younger age brackets than in older; however, all age brackets demonstrated higher rates than controls. This observation was consistent when analyzing the feature importance extracted from the ML models. Kissella et al. (2012) highlighted an increase in self-reported vascular risk factors among young people [32]. A recent European study demonstrated that smoking, dyslipidemia, and hypertension were the most common risk factors in people under 49 years of age with IS, versus coronary artery disease, atrial fibrillation, and diabetes, which were more characteristic of those over the age of 61 [33]. Overall, the reason why young people are suffering an increasing number of strokes remains unclear. An Australian study identified “cryptogenic” as the listed etiology in nearly half of the strokes occurring in patients aged 18–50 years, compared to only 18.1% in those over the age of 61 [34]. In our cohort, some other well-known vascular risk factors, such as obesity and family history of stroke or MI, remained high across all age brackets. It is conceivable that younger patients with vascular risk factors may be exponentially more at risk of having a stroke if their presentation is compounded by rare etiologies that are relatively less frequent in older individuals, such as migraine, hypercoagulable states, and PFO; however, the reason for the increasing prevalence of young stroke is yet to be elucidated.

### 4.3. Rarer Stroke Etiologies and Non-Traditional Risk Factors 

Interestingly, some known risk factors for stroke did not differ significantly between cases and controls in both males and females across all or most age brackets. These risk factors included elevated BMI, current smoking, cancer, rheumatic disease, alcohol abuse/dependence, drug abuse/dependence, and GCA. Some of the lack of difference observed in these risk factors may be related to the way variables were captured; for example, cocaine use is a known risk factor for stroke secondary to vasospasm, Ref. [35] but this may have been diluted when abuses of all drugs were condensed into a single variable. The fact that rates of smoking did not differ significantly between cases and controls is not clear; however, that could be related to better reporting of smoking status in EHR and an overall decrease in rates of smoking [36,37].

Anxiety and mood disorders (depression and bipolar disorder) were included in our analysis, given the increasing data supporting the association between mental illness and an increased risk of stroke [21,22]. For women, there was no difference in mood disorder prevalence between cases and controls; however, for men, both pre-index IS mood disorders and anxiety disorder had a significantly higher prevalence in those with stroke compared to control. This is also observed when analyzing the feature importance extracted from the ML models (Figure 3). While this finding does not imply causation, it highlights that psychological distress may play more of a role in stroke than previously thought. While there is good evidence surrounding post-stroke depression, Ref. [38] there is less information on how having a mood disorder may pre-dispose patients to stroke, particularly if the respective psychiatric diagnosis affects patients’ ability to seek out and engage with health-promotion efforts, which in turn highlights the importance of personalized and targeted management in stroke-prevention efforts. 

### 4.4. Gender, Genetic, and Environment Differences 

Several other non-age-related factors may play into the prevalence of “young strokes”, including environment and sex. Some studies have suggested that individuals residing in rural areas have worse stroke outcomes than in urban areas, and are plagued by more stroke risk factors [39]. There is a relative lack of data on young people with stroke in rural settings, making the present investigation based on a rural region in the United States a unique source of information. These patients may be unintentionally excluded from primary prevention efforts due to a lack of access to services or the misconception that their risk of stroke is limited due to their age [40,41].

Female “young stroke” is unique, given fluctuant hormone levels present in women during the peri-menopausal stage [42]. A European study identified dyslipidemia (51.6% in males versus 37.9% in females, *p* < 0.001), smoking (54.3% in males versus 41.4% in females, *p* < 0.001), and coronary heart disease (7.8% in males versus 3.8% in females, *p* < 0.001) as examples of risk factors that differed significantly between genders in patients with stroke, who were under the age of 49 [33]. There was no absolute sex difference in this cohort in terms of family history of stroke, hypertension, past transient ischemic attack (TIA), diabetes, peripheral arterial disease (PAD), AF, and heart failure (HF) [33]. However, many women will not have undergone menopause at age 49. A different pattern of gender difference might have merged in the 50–60 years old category, had they been included in the study [42,43]. Lisabeth et al. (2010) identified early menopause as a significant risk factor for stroke in women after the age of 60; however, limited data exist to suggest how the peri-menopausal risk factor profile differs from pre-menopausal women, and men [44]. 

There is a growing body of research looking at a genetic predisposition to stroke. Genetic causes of stroke may disproportionately affect young people. Interestingly a recent large-scale meta-analysis genome-wide association study (GWAS) identified the A1 blood haplotype variation at the *ABO* locus to be associated with early-onset IS compared to later-onset strokes [7]. The mechanism of this is hypothesized to be related to thrombosis rather than atherosclerosis. In that GWAS study, the age cut-point to define young stroke was 59 years for male and female patients.

### 4.5. Study Limitations, Strengths, and Future Directions

This study has several limitations, and the results must be interpreted within these constraints. The EHR data used in model development were rich and comprehensive; however, there is an inherent noise associated with EHR, including selection bias. The patient population is based on the regional demographics in central and northeast Pennsylvania, a predominantly Caucasian patient population. This study also had several strengths: using a large cohort from an integrated healthcare system and a data-driven strategy coupled with supervised modeling to corroborate the findings. 

When considering sex as a biological variable, the age for defining young stroke is also important. We have shown that a more precise definition can be reached if the analysis is done in parallel for male and female patients. Improved patient stratification for future investigations is critical in improving care and management for patients suffering a stroke at a younger age. Given the low proportion of strokes experienced by younger patients at any given teaching hospital, a meta-analysis may be the only way to truly elucidate what factors are driving the increasing stroke prevalence in this age group, and that is why having a consensus on defining “young stroke” is an essential first step. 

## 5. Conclusions

This is the first study to leverage large data and an innovative data-driven strategy to identify the most appropriate age to identify young male and female IS patients. Our data suggest that younger patients presented higher rates of traditional, as well as rare, risk factors than controls. In comparison, older patients displayed higher rates of traditional risk factors. A more precise cut-point in defining young stroke is 54 years for women and 52 years for men in our, mainly rural, population in central Pennsylvania. Future validation studies should include more diverse populations.

## Data Availability

The data presented in this study are available on request from the corresponding author. The data are not publicly available due to institutional policies requiring data-sharing agreement.

## Supplementary Materials

The following supporting information can be downloaded at: https://www.mdpi.com/article/10.3390/jcm12072600/s1.
