# Supplementary material for: Defining the Age of Young Ischemic Stroke Using Data-Driven Approaches
Source: J Clin Med. 2023 Mar 30;12(7):2600. doi: 10.3390/jcm12072600 (PMC10095415; doi:10.3390/jcm12072600)
Supplement: Supplementary file 1 [file jcm-12-02600-s001.zip › jcm-2267660-supplementary.pdf]

SUPPLEMETNAL MATERIAL FOR

Define the Age of Young Ischemic Stroke Using Data-Driven Approaches

Contents

Table S1. TRIPOD checklist. 2

Table S2. Variables and the respective time frame used in the second 4

Table S3. ICD-9-CM/ICD-10-CM Codes used to ischemic stroke and risk factors/comorbidities 5

Table S4. List of variables and the respective counts and percentages in different female groups based on the different age brackets. ** p<0.05 between cases and control for the age bracket.* 13

Table S5. List of variables and the respective counts and percentages in different male groups based on the different age brackets. ** p<0.05 between cases and control for the age bracket.* 14

Table S6. The best model for each age bracket for males and females, as well as optimized model parameters. 16

Table S7. Studies on young stroke patients with the respective findings and the age cut-point used. 17

Table S8. Model AUROC and Accuracy for all the models. 19

Figure S1. Model performance (AUROC and Accuracy) for different models. Performance measures for all the models are listed in Table S8. 21

Table S1. TRIPOD checklist.

Section/Topic			Checklist Item	PAGE
**Title and abstract**				
Title	1	D;V	Identify the study as developing and/or validating a multivariable prediction model, the target population, and the outcome to be predicted.	1
Abstract	2	D;V	Provide a summary of objectives, study design, setting, participants, sample size, predictors, outcome, statistical analysis, results, and conclusions.	1
**Introduction**				
Background and objectives	3a	D;V	Explain the medical context (including whether diagnostic or prognostic) and rationale for developing or validating the multivariable prediction model, including references to existing models.	1-2
	3b	D;V	Specify the objectives, including whether the study describes the development or validation of the model or both.	2
**Methods**				
Source of data	4a	D;V	Describe the study design or source of data (e.g., randomized trial, cohort, or registry data), separately for the development and validation data sets, if applicable.	2
	4b	D;V	Specify the key study dates, including start of accrual; end of accrual; and, if applicable, end of follow-up.	3
Participants	5a	D;V	Specify key elements of the study setting (e.g., primary care, secondary care, general population) including number and location of centres.	3
	5b	D;V	Describe eligibility criteria for participants.	3
	5c	D;V	Give details of treatments received, if relevant.	-
Outcome	6a	D;V	Clearly define the outcome that is predicted by the prediction model, including how and when assessed.	3
	6b	D;V	Report any actions to blind assessment of the outcome to be predicted.	-
Predictors	7a	D;V	Clearly define all predictors used in developing or validating the multivariable prediction model, including how and when they were measured.	3
	7b	D;V	Report any actions to blind assessment of predictors for the outcome and other predictors.	3
Sample size	8	D;V	Explain how the study size was arrived at.	3
Missing data	9	D;V	Describe how missing data were handled (e.g., complete-case analysis, single imputation, multiple imputation) with details of any imputation method.	3
Statistical analysis methods	10a	D	Describe how predictors were handled in the analyses.	3-4
	10b	D	Specify type of model, all model-building procedures (including any predictor selection), and method for internal validation.	3-4
	10c	V	For validation, describe how the predictions were calculated.	3-4
	10d	D;V	Specify all measures used to assess model performance and, if relevant, to compare multiple models.	
	10e	V	Describe any model updating (e.g., recalibration) arising from the validation, if done.	-
Risk groups	11	D;V	Provide details on how risk groups were created, if done.	3-4
Development vs. validation	12	V	For validation, identify any differences from the development data in setting, eligibility criteria, outcome, and predictors.	-
**Results**				
Participants	13a	D;V	Describe the flow of participants through the study, including the number of participants with and without the outcome and, if applicable, a summary of the follow-up time. A diagram may be helpful.	4
	13b	D;V	Describe the characteristics of the participants (basic demographics, clinical features, available predictors), including the number of participants with missing data for predictors and outcome.	4-5
	13c	V	For validation, show a comparison with the development data of the distribution of important variables (demographics, predictors and outcome).	-
Model development	14a	D	Specify the number of participants and outcome events in each analysis.	6-7
	14b	D	If done, report the unadjusted association between each candidate predictor and outcome.	-

Model specification	15a	D	Present the full prediction model to allow predictions for individuals (i.e., all regression coefficients, and model intercept or baseline survival at a given time point).	6-7
	15b	D	Explain how to use the prediction model.	6-7
Model performance	16	D;V	Report performance measures (with CIs) for the prediction model.	
Model-updating	17	V	If done, report the results from any model updating (i.e., model specification, model performance).	-
**Discussion**				
Limitations	18	D;V	Discuss any limitations of the study (such as nonrepresentative sample, few events per predictor, missing data).	9
Interpretation	19a	V	For validation, discuss the results with reference to performance in the development data, and any other validation data.	-
	19b	D;V	Give an overall interpretation of the results, considering objectives, limitations, results from similar studies, and other relevant evidence.	8-9
Implications	20	D;V	Discuss the potential clinical use of the model and implications for future research.	9-10
**Other information**				
Supplementary information	21	D;V	Provide information about the availability of supplementary resources, such as study protocol, Web calculator, and data sets.	3, 10
Funding	22	D;V	Give the source of funding and the role of the funders for the present study.	10

*Items relevant only to the development of a prediction model are denoted by D, items relating solely to a validation of a prediction model are denoted by V, and items relating to both are denoted D;V. We recommend using the TRIPOD Checklist in conjunction with the TRIPOD Explanation and Elaboration document.

Table S2. Variables and the respective time frame used in the second

VARIABLE		EXTRACTION TIME FRAME
ARTERIOPATHIES	Vasculitis	3-month buffer
	Fibromuscular dysplasia	Lifetime
	Reversible cerebral vasoconstriction syndrome	3-month buffer
	Moyamoya	Lifetime
	Sickle cell disease	Lifetime
CHRONIC SYSTEMIC DISORDERS	Chronic liver disease	At index stroke
	Chronic lung disease	At index stroke
	Chronic kidney disease	At index stroke
	Cirrhosis	3-month buffer
	End stage renal disease	At index stroke
HYPERCOAGULABLE STATE		3-month buffer
ATRIAL FIBRILLATION OR FLUTTER (AF)		3-month buffer
ALCOHOL DEPENDENCE OR ABUSE		At index stroke
CONGESTIVE HEART FAILURE (CHF)		At index stroke
DIABETES MELLITUS (DM)		At index stroke
DRUG DEPENDENCE AND ABUSE		At index stroke
FAMILY HISTORY OF HEART DISEASE AND/OR FAMILY HISTORY OF STROKE (FAMILY HISTORY)		At index stroke
MYOCARDIAL INFARCTION (MI)		At index stroke
PERIPHERAL VASCULAR DISEASE (PVD)		3-month buffer
PATENT FORAMEN OVALE (PFO)		Lifetime
SMOKING STATUS		At index stroke
ELEVATED BODY MASS INDEX (BMI)		At index stroke
CERVICOCEPHALIC ARTERIAL DISSECTION		At index stroke
NEOPLASM		At index stroke
RHEUMATIC DISEASE		3-month buffer
MIGRAINE		At index stroke
TEMPORAL ARTERITIS / GIANT CELL ARTERITIS (GCA)		3-month buffer
HYPERTENSION		3-month buffer
DYSLIPIDEMIA		At index stroke
MOOD DISORDER (DEPRESSION, BIPOLAR)		At index stroke
ANXIETY DISORDER		At index stroke

Table S3. ICD-9-CM/ICD-10-CM Codes used to ischemic stroke and risk factors/comorbidities

VARIABLE NAME	VARIABLE TYPE	UNIT/ FORMAT/ LEVELS	VARIABLE DESCRIPTION	ICD-9-CM CODE	ICD-10-CM CODE
**PATIENT ID**	String	Format: PT#####	Unique ID assigned to de-identified patients	n/a	n/a
**PATIENT SEX/GENDER**	Categorical	Levels:<br>Female<br>Male	Patient's Sex/Gender as recorded in patient's Electronic Health Records (EHR).	n/a	n/a
**INDEX STROKE DATE**	Date	YYYY-MM-DD	Date of first encounter at Geisinger in which:<br>a) primary diagnosis was ischemic stroke, and<br>b) MRI was available in the encounter, and<br>c) length of stay was at least 24 hours	n/a	n/a
**ICD CODE AT INDEX STROKE DATE**	String		ICD-9-CM or ICD-10-CM codes used for the primary diagnosis	433.01, 433.11, 433.21, 433.31, 433.81, 433.91, 434.01, 434.11, 434.91	I63, I63.0, I63.00, I63.01, I63.011, I63.012, I63.013, I63.019, I63.02, I63.03, I63.031, I63.032, I63.033, I63.039, I63.09, I63.1, I63.10, I63.11, I63.111, I63.112, I63.113, I63.119, I63.12, I63.13, I63.131, I63.132, I63.133, I63.139, I63.19, I63.2, I63.20, I63.21, I63.211, I63.212, I63.213, I63.219, I63.22, I63.23, I63.231, I63.232, I63.233, I63.239, I63.29, I63.3, I63.30, I63.31, I63.311, I63.312, I63.313, I63.319, I63.32, I63.321, I63.322, I63.323, I63.329, I63.33, I63.331, I63.332, I63.333, I63.339, I63.34, I63.341, I63.342, I63.343, I63.349, I63.39, I63.4, I63.40, I63.41, I63.411, I63.412, I63.413, I63.419, I63.42, I63.421, I63.422, I63.423, I63.429, I63.43, I63.431, I63.432, I63.433, I63.439, I63.44, I63.441, I63.442, I63.443, I63.449, I63.49, I63.5, I63.50, I63.51, I63.511, I63.512, I63.513, I63.519, I63.52, I63.521, I63.522, I63.523, I63.529, I63.53, I63.531, I63.532, I63.533, I63.539, I63.54, I63.541, I63.542, I63.543, I63.549, I63.59, I63.6, I63.8, I63.9
**NAME OF THE ICD CODE AT INDEX STROKE DATE**	String		Corresponding primary diagnosis based on the ICD code used	n/a	n/a
**PATIENT'S AGE AT INDEX STROKE DATE**	Numerical	Unit: Years	Age of the patient at the Index Stroke Date; determined from Patient's Birthdate and Index Stroke Date<br><br>Comments: Changed to 89 if value greater than 89 as part of de-identification	n/a	n/a
**ISCHEMIC STROKE**	Categorical	Levels:<br>0 = Absent<br>1 = Present	Patients with ischemic stroke were included in study	433.01, 433.11, 433.21, 433.31, 433.81, 433.91, 434.01, 434.11, 434.91	I63, I63.0, I63.00, I63.01, I63.011, I63.012, I63.013, I63.019, I63.02, I63.03, I63.031, I63.032, I63.033, I63.039, I63.09, I63.1, I63.10, I63.11, I63.111, I63.112, I63.113, I63.119, I63.12, I63.13, I63.131, I63.132, I63.133, I63.139, I63.19, I63.2, I63.20, I63.21, I63.211, I63.212, I63.213, I63.219, I63.22, I63.23, I63.231, I63.232, I63.233, I63.239, I63.29, I63.3, I63.30, I63.31, I63.311, I63.312, I63.313, I63.319, I63.32, I63.321, I63.322, I63.323, I63.329, I63.33, I63.331, I63.332, I63.333, I63.339, I63.34, I63.341, I63.342, I63.343, I63.349, I63.39, I63.4, I63.40, I63.41, I63.411, I63.412, I63.413, I63.419, I63.42, I63.421, I63.422, I63.423, I63.429, I63.43, I63.431, I63.432, I63.433, I63.439, I63.44, I63.441, I63.442, I63.443, I63.449, I63.49, I63.5, I63.50, I63.51, I63.511, I63.512, I63.513, I63.519, I63.52, I63.521, I63.522, I63.523, I63.529, I63.53, I63.531, I63.532, I63.533, I63.539, I63.54, I63.541, I63.542, I63.543, I63.549, I63.59, I63.6, I63.8, I63.9

**ATRIAL FIBRILLATION / FLUTTER (AFIB/FLUTTER)**	Categorical	Levels:<br>0 = Absent<br>1 = Present	Presence of atrial fibrillation or flutter at time of index stroke or within 3 months of index stroke	427.3, 427.31, 427.32	I48, I48.0, I48.1, I48.11, I48.19, I48.2, I48.20, I48.21, I48.9, I48.91, I48.3, I48.4, I48.92
**ALCOHOL DEPENDANCE OR ABUSE**	Categorical	Levels:<br>0 = Absent<br>1 = Present	Presence of alcohol dependence of abuse at time of index stroke	305.0, 305.00, 305.01, 305.02, 305.03	F10.1, F10.10, F10.11, F10.12, F10.120, F10.121, F10.129, F10.14, F10.15, F10.150, F10.151, F10.159, F10.18, F10.180, F10.181, F10.182, F10.188, F10.19, F10.2, F10.20, F10.21, F10.22, F10.220, F10.221, F10.229, F10.23, F10.230, F10.231, F10.232, F10.239, F10.24, F10.25, F10.250, F10.251, F10.259, F10.26, F10.27, F10.28, F10.280, F10.281, F10.282, F10.288, F10.29, F10.9, F10.92, F10.920, F10.921, F10.929, F10.94, F10.95, F10.950, F10.951, F10.959, F10.96, F10.97, F10.98, F10.980, F10.981, F10.982, F10.988, F10.99
**BODY MASS INDEX (BMI)**	Continuous imputed and dichotomized	Levels:<br>0 = BMI <24<br>1 = BMI >/= 24	Overweight (BMI >24) at index stroke	n/a	n/a
**CENTRAL NERVOUS SYSTEM (CNS) NEOPLASM**	Categorical	Levels:<br>0 = Absent<br>1 = Present	Presence of a brain tumor at time of index stroke	191, 191.0, 191.1, 191.2, 191.3, 191.4, 191.5, 191.6, 191.7, 191.8, 191.9, 225, 225.0, 225.1, 225.2, 225.3, 225.4, 225.8, 225.9, 239.6, 198.3	C71, C71.0, C71.1, C71.2, C71.3, C71.4, C71.5, C71.6, C71.7, C71.8, C71.9, D33, D33.0, D33.1, D33.2, D33.3, D33.4, D33.7, D33.9, C79.3, C79.31, C79.32
**CERVICAL ARTERY DISSECTION**	Categorical	Levels:<br>0 = Absent<br>1 = Present	Presence of cervicocephalic arterial dissection at time of index stroke	443.21, 443.24	I77.71, I77.74
**HEART FAILURE**	Categorical	Levels:<br>0 = Absent<br>1 = Present	Presence of heart failure at time of index stroke	402.01, 402.11, 402.91, 404.01, 404.03, 404.11, 404.13, 404.91, 404.93, 428, 428.0, 428.1, 428.2, 428.20, 428.21, 428.22, 428.23, 428.3, 428.30, 428.31, 428.32, 428.33, 428.4, 428.40, 428.41, 428.42, 428.43, 428.9	I11.0, I13.0, I13.2, I50, I50.1, I50.2, I50.20, I50.21, I50.22, I50.23, I50.3, I50.30, I50.31, I50.32, I50.33, I50.4, I50.40, I50.41, I50.42, I50.43, I50.8, I50.81, I50.810, I50.811, I50.812, I50.813, I50.814, I50.82, I50.83, I50.84, I50.89, I50.9
**DIABETES MELLITUS (DM)**	Categorical	Levels:<br>0 = Absent<br>1 = Present	Presence of diabetes at time of index stroke	249, 249.0, 249.00, 249.01, 249.1, 249.10, 249.11, 249.2, 249.20, 249.21, 249.3, 249.30, 249.31, 249.4, 249.40, 249.41, 249.5, 249.50, 249.51, 249.6, 249.60, 249.61, 249.7, 249.70, 249.71, 249.8, 249.80, 249.81, 249.9, 249.90, 249.91, 250, 250.0, 250.00, 250.01, 250.02, 250.03, 250.1, 250.10, 250.11, 250.12, 250.13, 250.2, 250.20, 250.21, 250.22, 250.23, 250.3, 250.30, 250.31, 250.32, 250.33, 250.4, 250.40, 250.41, 250.42, 250.43, 250.5, 250.50, 250.51, 250.52, 250.53, 250.6, 250.60, 250.61, 250.62, 250.63, 250.7, 250.70, 250.71, 250.72, 250.73, 250.8, 250.80, 250.81, 250.82, 250.83, 250.9, 250.90, 250.91, 250.92, 250.93, 357.2, 362.01, 362.02, 362.03, 362.04, 362.05, 362.06, 362.07, 366.41	E10 , E10.1 , E10.10 , E10.11 , E10.2 , E10.21 , E10.22 , E10.29 , E10.3 , E10.31 , E10.311 , E10.319 , E10.32 , E10.321 , E10.3211 , E10.3212 , E10.3213 , E10.3219 , E10.329 , E10.3291 , E10.3292 , E10.3293 , E10.3299 , E10.33 , E10.331 , E10.3311 , E10.3312 , E10.3313 , E10.3319 , E10.339 , E10.3391 , E10.3392 , E10.3393 , E10.3399 , E10.34 , E10.341 , E10.3411 , E10.3412 , E10.3413 , E10.3419 , E10.349 , E10.3491 , E10.3492 , E10.3493 , E10.3499 , E10.35 , E10.351 , E10.3511 , E10.3512 , E10.3513 , E10.3519 , E10.352 , E10.3521 , E10.3522 , E10.3523 , E10.3529 , E10.353 , E10.3531 , E10.3532 , E10.3533 , E10.3539 , E10.354 , E10.3541 , E10.3542 , E10.3543 , E10.3549 , E10.355 , E10.3551 , E10.3552 , E10.3553 , E10.3559 , E10.359 , E10.3591 , E10.3592 , E10.3593 , E10.3599 , E10.36 , E10.37 , E10.37X1 , E10.37X2 , E10.37X3 , E10.37X9 , E10.39 , E10.4 , E10.40 , E10.41 , E10.42 , E10.43 , E10.44 , E10.49 , E10.5 , E10.51 , E10.52 , E10.59 , E10.6 , E10.61 , E10.610 , E10.618 , E10.62 , E10.620 , E10.621 , E10.622 , E10.628 , E10.63 , E10.630 , E10.638 , E10.64 , E10.641 , E10.649 , E10.65 , E10.69 , E10.8 , E10.9 , E11 , E11.0 , E11.00 , E11.01 , E11.1 , E11.10 , E11.11 , E11.2 , E11.21 , E11.22 , E11.29 , E11.3 , E11.31 , E11.311 , E11.319 , E11.32 , E11.321 , E11.3211 , E11.3212 , E11.3213 , E11.3219 , E11.329 , E11.3291 , E11.3292 , E11.3293 , E11.3299 , E11.33 , E11.331 , E11.3311 , E11.3312 , E11.3313 , E11.3319 , E11.339 , E11.3391 , E11.3392 , E11.3393 , E11.3399 , E11.34 , E11.341 , E11.3411 , E11.3412 , E11.3413 , E11.3419 , E11.349 , E11.3491 , E11.3492 , E11.3493 , E11.3499 , E11.35 , E11.351 , E11.3511 , E11.3512 , E11.3513 , E11.3519 , E11.352 , E11.3521 , E11.3522 , E11.3523 , E11.3529 , E11.353 , E11.3531 , E11.3532 , E11.3533 , E11.3539 , E11.354 , E11.3541 , E11.3542 , E11.3543 , E11.3549 , E11.355 , E11.3551 , E11.3552 , E11.3553 , E11.3559 , E11.359 , E11.3591 , E11.3592 , E11.3593 , E11.3599 , E11.36 , E11.37 , E11.37X1 , E11.37X2 , E11.37X3 , E11.37X9 , E11.39 , E11.4 , E11.40 , E11.41 , E11.42 , E11.43 , E11.44 , E11.49 , E11.5 , E11.51 , E11.52 , E11.59 , E11.6 , E11.61 , E11.610 , E11.618 , E11.62 , E11.620 , E11.621 , E11.622 , E11.628 , E11.63 , E11.630 , E11.638 , E11.64 , E11.641 , E11.649 , E11.65 , E11.69 , E11.8 , E11.9 , E08 , E08.0 , E08.00 , E08.01 , E08.1 , E08.10 , E08.11 , E08.2 , E08.21 , E08.22 , E08.29 , E08.3 , E08.31 , E08.311 , E08.319 , E08.32 , E08.321 , E08.3211 , E08.3212 , E08.3213 , E08.3219 , E08.329 , E08.3291 , E08.3292 , E08.3293 , E08.3299 , E08.33 , E08.331 , E08.3311 , E08.3312 , E08.3313 , E08.3319 , E08.339 , E08.3391 , E08.3392 , E08.3393 , E08.3399 , E08.34 , E08.341 , E08.3411 , E08.3412 , E08.3413 , E08.3419 , E08.349 , E08.3491 , E08.3492 , E08.3493 , E08.3499 , E08.35 , E08.351 , E08.3511 , E08.3512 , E08.3513 , E08.3519 , E08.352 , E08.3521 , E08.3522 , E08.3523 , E08.3529 , E08.353 , E08.3531 , E08.3532 , E08.3533 , E08.3539 , E08.354 , E08.3541 , E08.3542 , E08.3543 , E08.3549 , E08.355 , E08.3551 , E08.3552 , E08.3553 , E08.3559 , E08.359 , E08.3591 , E08.3592 ,

					E08.3593 , E08.3599 , E08.36 , E08.37 , E08.37X1 , E08.37X2 , E08.37X3 , E08.37X9 , E08.39 , E08.4 , E08.40 , E08.41 , E08.42 , E08.43 , E08.44 , E08.49 , E08.5 , E08.51 , E08.52 , E08.59 , E08.6 , E08.61 , E08.610 , E08.618 , E08.62 , E08.620 , E08.621 , E08.622 , E08.628 , E08.63 , E08.630 , E08.638 , E08.64 , E08.641 , E08.649 , E08.65 , E08.69 , E08.8 , E08.9 , E09 , E09.0 , E09.00 , E09.01 , E09.1 , E09.10 , E09.11 , E09.2 , E09.21 , E09.22 , E09.29 , E09.3 , E09.31 , E09.311 , E09.319 , E09.32 , E09.321 , E09.3211 , E09.3212 , E09.3213 , E09.3219 , E09.329 , E09.3291 , E09.3292 , E09.3293 , E09.3299 , E09.33 , E09.331 , E09.3311 , E09.3312 , E09.3313 , E09.3319 , E09.339 , E09.3391 , E09.3392 , E09.3393 , E09.3399 , E09.34 , E09.341 , E09.3411 , E09.3412 , E09.3413 , E09.3419 , E09.349 , E09.3491 , E09.3492 , E09.3493 , E09.3499 , E09.35 , E09.351 , E09.3511 , E09.3512 , E09.3513 , E09.3519 , E09.352 , E09.3521 , E09.3522 , E09.3523 , E09.3529 , E09.353 , E09.3531 , E09.3532 , E09.3533 , E09.3539 , E09.354 , E09.3541 , E09.3542 , E09.3543 , E09.3549 , E09.355 , E09.3551 , E09.3552 , E09.3553 , E09.3559 , E09.359 , E09.3591 , E09.3592 , E09.3593 , E09.3599 , E09.36 , E09.37 , E09.37X1 , E09.37X2 , E09.37X3 , E09.37X9 , E09.39 , E09.4 , E09.40 , E09.41 , E09.42 , E09.43 , E09.44 , E09.49 , E09.5 , E09.51 , E09.52 , E09.59 , E09.6 , E09.61 , E09.610 , E09.618 , E09.62 , E09.620 , E09.621 , E09.622 , E09.628 , E09.63 , E09.630 , E09.638 , E09.64 , E09.641 , E09.649 , E09.65 , E09.69 , E09.8 , E09.9 , E13 , E13.0 , E13.00 , E13.01 , E13.1 , E13.10 , E13.11 , E13.2 , E13.21 , E13.22 , E13.29 , E13.3 , E13.31 , E13.311 , E13.319 , E13.32 , E13.321 , E13.3211 , E13.3212 , E13.3213 , E13.3219 , E13.329 , E13.3291 , E13.3292 , E13.3293 , E13.3299 , E13.33 , E13.331 , E13.3311 , E13.3312 , E13.3313 , E13.3319 , E13.339 , E13.3391 , E13.3392 , E13.3393 , E13.3399 , E13.34 , E13.341 , E13.3411 , E13.3412 , E13.3413 , E13.3419 , E13.349 , E13.3491 , E13.3492 , E13.3493 , E13.3499 , E13.35 , E13.351 , E13.3511 , E13.3512 , E13.3513 , E13.3519 , E13.352 , E13.3521 , E13.3522 , E13.3523 , E13.3529 , E13.353 , E13.3531 , E13.3532 , E13.3533 , E13.3539 , E13.354 , E13.3541 , E13.3542 , E13.3543 , E13.3549 , E13.355 , E13.3551 , E13.3552 , E13.3553 , E13.3559 , E13.359 , E13.3591 , E13.3592 , E13.3593 , E13.3599 , E13.36 , E13.37 , E13.37X1 , E13.37X2 , E13.37X3 , E13.37X9 , E13.39 , E13.4 , E13.40 , E13.41 , E13.42 , E13.43 , E13.44 , E13.49 , E13.5 , E13.51 , E13.52 , E13.59 , E13.6 , E13.61 , E13.610 , E13.618 , E13.62 , E13.620 , E13.621 , E13.622 , E13.628 , E13.63 , E13.630 , E13.638 , E13.64 , E13.641 , E13.649 , E13.65 , E13.69 , E13.8 , E13.9
**DRUG DEPENDANCE OF ABUSE**	Categorical	Levels:<br>0 = Absent<br>1 = Present	Presence of drug abuse or dependence at time of index stroke<br><br>Opioids<br>Cannabis<br>Cocaine<br>Other drugs	1.<br>304.0, 304.00, 304.01, 304.02, 304.03, 304.7, 304.70, 304.71, 304.72, 304.73, 305.5, 305.50, 305.51, 305.52, 305.53<br><br>2.<br>304.3, 304.30, 304.31, 304.32, 304.33, 305.2, 305.20, 305.21, 305.22, 305.23<br><br>3.<br>304.2, 304.20, 304.21, 304.22, 304.23, 305.6, 305.60, 305.61, 305.62, 305.63<br><br>4.<br>304.1, 304.10, 304.11, 304.12, 304.13, 304.4, 304.40, 304.41, 304.42, 304.43, 304.5, 304.50, 304.51, 304.52, 304.53, 304.6, 304.60, 304.61, 304.62, 304.63, 304.8, 304.80, 304.81, 304.82, 304.83, 304.9, 304.90, 304.91, 304.92, 304.93, 305.3, 305.30, 305.31, 305.32, 305.33, 305.4, 305.40, 305.41, 305.42, 305.43, 305.7, 305.70, 305.71, 305.72, 305.73, 305.8, 305.80, 305.81, 305.82, 305.83, 305.9, 305.90, 305.91, 305.92, 305.93	1.<br>F11, F11.1, F11.10, F11.11, F11.12, F11.120, F11.121, F11.122, F11.129, F11.14, F11.15, F11.150, F11.151, F11.159, F11.18, F11.181, F11.182, F11.188, F11.19, F11.2, F11.20, F11.21, F11.22, F11.220, F11.221, F11.222, F11.229, F11.23, F11.24, F11.25, F11.250, F11.251, F11.259, F11.28, F11.281, F11.282, F11.288, F11.29, F11.9, F11.90, F11.92, F11.920, F11.921, F11.922, F11.929, F11.93, F11.94, F11.95, F11.950, F11.951, F11.959, F11.98, F11.981, F11.982, F11.988, F11.99<br><br>2.<br>F12, F12.1, F12.10, F12.11, F12.12, F12.120, F12.121, F12.122, F12.129, F12.15, F12.150, F12.151, F12.159, F12.18, F12.180, F12.188, F12.19, F12.2, F12.20, F12.21, F12.22, F12.220, F12.221, F12.222, F12.229, F12.23, F12.25, F12.250, F12.251, F12.259, F12.28, F12.280, F12.288, F12.29, F12.9, F12.90, F12.92, F12.920, F12.921, F12.922, F12.929, F12.93, F12.95, F12.950, F12.951, F12.959, F12.98, F12.980, F12.988, F12.99<br><br>3.<br>F14, F14.1, F14.10, F14.11, F14.12, F14.120, F14.121, F14.122, F14.129, F14.14, F14.15, F14.150, F14.151, F14.159, F14.18, F14.180, F14.181, F14.182, F14.188, F14.19, F14.2, F14.20, F14.21, F14.22, F14.220, F14.221, F14.222, F14.229, F14.23, F14.24, F14.25, F14.250, F14.251, F14.259, F14.28, F14.280, F14.281, F14.282, F14.288, F14.29, F14.9, F14.90, F14.92, F14.920, F14.921, F14.922, F14.929, F14.94, F14.95, F14.950, F14.951, F14.959, F14.98, F14.980, F14.981, F14.982, F14.988, F14.99<br><br>4.<br>F13, F13.1, F13.10, F13.11, F13.12, F13.120, F13.121, F13.129, F13.14, F13.15, F13.150, F13.151, F13.159, F13.18, F13.180, F13.181, F13.182, F13.188, F13.19, F13.2, F13.20, F13.21, F13.22, F13.220, F13.221, F13.229, F13.23, F13.230, F13.231, F13.232, F13.239, F13.24, F13.25, F13.250, F13.251, F13.259, F13.26, F13.27, F13.28, F13.280, F13.281, F13.282, F13.288, F13.29, F13.9, F13.90, F13.92, F13.920, F13.921, F13.929, F13.93, F13.930, F13.931, F13.932, F13.939, F13.94, F13.95, F13.950, F13.951, F13.959, F13.96, F13.97, F13.98, F13.980, F13.981, F13.982, F13.988, F13.99, F15, F15.1, F15.10, F15.11, F15.12, F15.120, F15.121, F15.122, F15.129, F15.14, F15.15, F15.150, F15.151, F15.159, F15.18, F15.180, F15.181, F15.182, F15.188, F15.19, F15.2,

					F15.20, F15.21, F15.22, F15.220, F15.221, F15.222, F15.229, F15.23, F15.24, F15.25, F15.250, F15.251, F15.259, F15.28, F15.280, F15.281, F15.282, F15.288, F15.29, F15.9, F15.90, F15.92, F15.920, F15.921, F15.922, F15.929, F15.93, F15.94, F15.95, F15.950, F15.951, F15.959, F15.98, F15.980, F15.981, F15.982, F15.988, F15.99, F16, F16.1, F16.10, F16.11, F16.12, F16.120, F16.121, F16.122, F16.129, F16.14, F16.15, F16.150, F16.151, F16.159, F16.18, F16.180, F16.183, F16.188, F16.19, F16.2, F16.20, F16.21, F16.22, F16.220, F16.221, F16.229, F16.24, F16.25, F16.250, F16.251, F16.259, F16.28, F16.280, F16.283, F16.288, F16.29, F16.9, F16.90, F16.92, F16.920, F16.921, F16.929, F16.94, F16.95, F16.950, F16.951, F16.959, F16.98, F16.980, F16.983, F16.988, F16.99, F18, F18.1, F18.10, F18.11, F18.12, F18.120, F18.121, F18.129, F18.14, F18.15, F18.150, F18.151, F18.159, F18.17, F18.18, F18.180, F18.188, F18.19, F18.2, F18.20, F18.21, F18.22, F18.220, F18.221, F18.229, F18.24, F18.25, F18.250, F18.251, F18.259, F18.27, F18.28, F18.280, F18.288, F18.29, F18.9, F18.90, F18.92, F18.920, F18.921, F18.929, F18.94, F18.95, F18.950, F18.951, F18.959, F18.97, F18.98, F18.980, F18.988, F18.99, F19, F19.1, F19.10, F19.11, F19.12, F19.120, F19.121, F19.122, F19.129, F19.14, F19.15, F19.150, F19.151, F19.159, F19.16, F19.17, F19.18, F19.180, F19.181, F19.182, F19.188, F19.19, F19.2, F19.20, F19.21, F19.22, F19.220, F19.221, F19.222, F19.229, F19.23, F19.230, F19.231, F19.232, F19.239, F19.24, F19.25, F19.250, F19.251, F19.259, F19.26, F19.27, F19.28, F19.280, F19.281, F19.282, F19.288, F19.29, F19.9, F19.90, F19.92, F19.920, F19.921, F19.922, F19.929, F19.93, F19.930, F19.931, F19.932, F19.939, F19.94, F19.95, F19.950, F19.951, F19.959, F19.96, F19.97, F19.98, F19.980, F19.981, F19.982, F19.988, F19.99
**DYSLIPIDEMIA**	Categorical	Levels:<br>0 = Absent<br>1 = Present	Presence of dyslipidemia at time of index stroke	272.0, 272.1, 272.2, 272.3, 272.4, 272.5	E78.0, E78.00, E78.01, E78.1, E78.2, E78.3, E78.4, E78.41, E78.49, E78.5, E78.6
**FAMILY HISTORY OF STROKE OR MI**	Categorical	Levels:<br>0 = Absent<br>1 = Present	Recorded family history of either stroke or MI, or both, at any time in the EHR	n/a	n/a
**HYPERCOAGUABLE STATES**	Categorical	Levels:<br>0 = Absent<br>1 = Present	Presence of a hypercoagulable state at time of index stroke or within 3 months of index stroke	289.81, 289.82	D68.5, D68.51, D68.52, D68.59, D68.6, D68.61, D68.62, D68.69
**HYPERTENSION (HTN)**	Categorical	Levels:<br>0 = Absent<br>1 = Present	Presence of hypertension at time of index stroke	401, 401.0, 401.1, 401.9, 402, 402.0, 402.00, 402.01, 402.1, 402.10, 402.11, 402.9, 402.90, 402.91, 403, 403.0, 403.00, 403.01, 403.1, 403.10, 403.11, 403.9, 403.90, 403.91, 404, 404.0, 404.00, 404.01, 404.02, 404.03, 404.1, 404.10, 404.11, 404.12, 404.13, 404.9, 404.90, 404.91, 404.92, 404.93, 405, 405.0, 405.01, 405.09, 405.1, 405.11, 405.19, 405.9, 405.91, 405.99	I10, I11, I11.0, I11.9, I12, I12.0, I12.9, I13, I13.0, I13.1, I13.10, I13.11, I13.2, I15, I15.0, I15.1, I15.2, I15.8, I15.9, I16, I16.0, I16.1, I16.9
**MYOCARDIAL INFARCTION (MI)**	Categorical	Levels:<br>0 = Absent<br>1 = Present	Presence of MI at the time of index stroke	410, 410.0, 410.00, 410.01, 410.02, 410.1, 410.10, 410.11, 410.12, 410.2, 410.20, 410.21, 410.22, 410.3, 410.30, 410.31, 410.32, 410.4, 410.40, 410.41, 410.42, 410.5, 410.50, 410.51, 410.52, 410.6, 410.60, 410.61, 410.62, 410.7, 410.70, 410.71, 410.72, 410.8, 410.80, 410.81, 410.82, 410.9, 410.90, 410.91, 410.92, 412, 429.79	I21, I21.0, I21.01, I21.02, I21.09, I21.1, I21.11, I21.19, I21.2, I21.21, I21.29, I21.3, I21.4, I21.9, I21.A, I21.A1, I21.A9, I22, I22.0, I22.1, I22.2, I22.8, I22.9, I23, I23.0, I23.1, I23.2, I23.3, I23.4, I23.5, I23.6, I23.7, I23.8, I25.2
**MIGRAINE**	Categorical	Levels:<br>0 = Absent<br>1 = Present	Presence of a diagnosis of migraines at the time of index stroke	346, 346.0, 346.00, 346.01, 346.02, 346.03, 346.1, 346.10, 346.11, 346.12, 346.13, 346.2, 346.20, 346.21, 346.22, 346.23, 346.3, 346.30, 346.31, 346.32, 346.33, 346.4, 346.40, 346.41, 346.42, 346.43, 346.5, 346.50, 346.51, 346.52, 346.53, 346.7, 346.70, 346.71, 346.72, 346.73, 346.8, 346.80, 346.81, 346.82, 346.83, 346.9, 346.90, 346.91, 346.92, 346.93	G43, G43.0, G43.00, G43.001, G43.009, G43.01, G43.011, G43.019, G43.1, G43.10, G43.101, G43.109, G43.11, G43.111, G43.119, G43.4, G43.40, G43.401, G43.409, G43.41, G43.411, G43.419, G43.5, G43.50, G43.501, G43.509, G43.51, G43.511, G43.519, G43.7, G43.70, G43.701, G43.709, G43.71, G43.711, G43.719, G43.A, G43.A0, G43.A1, G43.B, G43.B0, G43.B1, G43.C, G43.C0, G43.C1, G43.D, G43.D0, G43.D1, G43.8, G43.80, G43.801, G43.809, G43.81, G43.811, G43.819, G43.82, G43.821, G43.829, G43.83, G43.831, G43.839, G43.9, G43.90, G43.901, G43.909, G43.91, G43.911, G43.919
**NON CNS NEOPLASM AT INDEX**	Categorical	Levels:<br>0 = Absent<br>1 = Present	Presence of a neoplasm (non -CNS) at the time of index stroke or within 3 months of index stroke	140.0, 140.1, 140.4, 140.5, 140.6, 140.8, 140.9, 141.0, 141.1, 141.2, 141.3, 141.4, 141.5, 141.6, 141.8, 141.9, 142.0, 142.1, 142.2, 142.8, 142.9, 143.0, 143.1, 143.8, 143.9, 144.0, 144.1, 144.8, 144.9, 145.0, 145.1, 145.2, 145.3, 145.4, 145.5, 145.6, 145.8, 145.9, 146.0, 146.1, 146.2, 146.3, 146.4, 146.5, 146.6, 146.7, 146.8, 146.9, 147.0, 147.1, 147.2, 147.3, 147.8, 147.9, 148.0, 148.1, 148.2, 148.3, 148.8, 148.9, 149.0, 149.8, 149.9, 150.0, 150.1, 150.2, 150.3, 150.4, 150.5, 150.8,	C00, C00.0, C00.1, C00.2, C00.3, C00.4, C00.5, C00.6, C00.8, C00.9, C01, C02, C02.0, C02.1, C02.2, C02.3, C02.4, C02.8, C02.9, C03, C03.0, C03.1, C03.9, C04, C04.0, C04.1, C04.8, C04.9, C05, C05.0, C05.1, C05.2, C05.8, C05.9, C06, C06.0, C06.1, C06.2, C06.8, C06.80, C06.89, C06.9, C07, C08, C08.0, C08.1, C08.9, C09, C09.0, C09.1, C09.8, C09.9, C10, C10.0, C10.1, C10.2, C10.3, C10.4, C10.8, C10.9, C11, C11.0, C11.1, C11.2, C11.3, C11.8, C11.9, C12, C13, C13.0, C13.1, C13.2, C13.8, C13.9, C14, C14.0, C14.2, C14.8, C15, C15.3, C15.4, C15.5, C15.8, C15.9, C16, C16.0, C16.1, C16.2, C16.3, C16.4, C16.5, C16.6, C16.8, C16.9, C17, C17.0, C17.1, C17.2, C17.3, C17.8, C17.9, C18, C18.0, C18.1, C18.2, C18.3, C18.4, C18.5, C18.6, C18.7, C18.8, C18.9, C19, C20, C21, C21.0, C21.1, C21.2, C21.8, C22, C22.0, C22.1, C22.2,

150.9, 151.0, 151.1, 151.2, 151.3, 151.4, 151.5, 151.6, 151.8, 151.9, 152.0, 152.1, 152.2, 152.3, 152.8, 152.9, 153, 153.0, 153.1, 153.2, 153.3, 153.4, 153.5, 153.6, 153.7, 153.8, 153.9, 154, 154.0, 154.1, 154.2, 154.3, 154.8, 155.0, 155.1, 155.2, 156.0, 156.1, 156.2, 156.8, 156.9, 157, 157.0, 157.1, 157.2, 157.3, 157.4, 157.8, 157.9, 158, 158.0, 158.8, 158.9, 159, 159.0, 159.1, 159.8, 159.9, 160.0, 160.1, 160.2, 160.3, 160.4, 160.5, 160.9, 161.0, 161.1, 161.2, 161.3, 161.8, 161.9, 162, 162.0, 162.2, 162.3, 162.4, 162.5, 162.8, 162.9, 163.0, 163.1, 163.8, 163.9, 164.0, 164.1, 164.2, 164.3, 164.8, 164.9, 165.8, 165.9, 170.0, 170.1, 170.2, 170.3, 170.4, 170.5, 170.6, 170.7, 170.8, 170.9, 171.0, 171.2, 171.3, 171.4, 171.5, 171.6, 171.7, 171.8, 171.9, 172.0, 172.1, 172.2, 172.3, 172.4, 172.5, 172.6, 172.7, 172.8, 172.9, 173.0, 173.00, 173.01, 173.02, 173.09, 173.1, 173.10, 173.11, 173.12, 173.19, 173.2, 173.20, 173.21, 173.22, 173.29, 173.3, 173.30, 173.31, 173.32, 173.39, 173.4, 173.40, 173.41, 173.42, 173.49, 173.5, 173.50, 173.51, 173.52, 173.59, 173.6, 173.60, 173.61, 173.62, 173.69, 173.7, 173.70, 173.71, 173.72, 173.79, 173.8, 173.80, 173.81, 173.82, 173.89, 173.9, 173.90, 173.91, 173.92, 173.99, 174, 174.0, 174.1, 174.2, 174.3, 174.4, 174.5, 174.6, 174.8, 174.9, 175.0, 175.9, 176.0, 176.1, 176.2, 176.3, 176.4, 176.8, 176.9, 179, 180.0, 180.1, 180.8, 180.9, 181, 182.0, 182.1, 182.8, 183, 183.0, 183.2, 183.4, 183.5, 183.8, 183.9, 184.0, 184.1, 184.2, 184.3, 184.4, 184.8, 184.9, 185, 186.0, 186.9, 187.1, 187.2, 187.3, 187.4, 187.6, 187.7, 187.8, 187.9, 188.0, 188.1, 188.2, 188.3, 188.4, 188.5, 188.6, 188.7, 188.8, 188.9, 189.0, 189.1, 189.2, 189.3, 189.4, 189.8, 189.9, 190.0, 190.1, 190.2, 190.3, 190.4, 190.5, 190.6, 190.7, 190.8, 190.9, 193, 194.0, 194.1, 194.3, 194.4, 194.5, 194.6, 194.8, 194.9, 195.0, 195.1, 195.2, 195.3, 195.4, 195.5, 195.8, 196.0, 196.1, 196.2, 196.3, 196.5, 196.6, 196.8, 196.9, 197.0, 197.1, 197.2, 197.3, 197.4, 197.5, 197.6, 197.7, 197.8, 198.0, 198.1, 198.2, 198.4, 198.5, 198.6, 198.7, 198.81, 198.82, 198.89, 199, 199.0, 199.1, 199.2, 200, 200.00, 200.01, 200.02, 200.03, 200.04, 200.05, 200.06, 200.08, 200.10, 200.11, 200.12, 200.13, 200.14, 200.15, 200.16, 200.18, 200.20, 200.21, 200.22, 200.23, 200.24, 200.27, 200.28, 200.30, 200.31, 200.32, 200.33, 200.34, 200.35, 200.37, 200.38, 200.40, 200.41, 200.42, 200.43, 200.44, 200.45, 200.47, 200.48, 200.50, 200.51, 200.53, 200.57, 200.58, 200.60, 200.61, 200.62, 200.63, 200.64, 200.65, 200.68, 200.70, 200.71, 200.72, 200.73, 200.74, 200.75, 200.77, 200.78, 200.8, 200.80, 200.81, 200.82, 200.83, 200.88, 201.00, 201.01, 201.10, 201.12, 201.20, 201.21, 201.25, 201.26, 201.40, 201.42, 201.43, 201.44, 201.45, 201.47, 201.48, 201.50, 201.51, 201.52, 201.53, 201.54, 201.58, 201.60, 201.61, 201.63, 201.64, 201.68, 201.70, 201.71, 201.72, 201.73, 201.74, 201.75, 201.78, 201.9, 201.90, 201.91, 201.92, 201.93, 201.94, 201.95, 201.96, 201.97, 201.98, 202.00, 202.01, 202.02, 202.03, 202.04, 202.05, 202.06, 202.07, 202.08, 202.10, 202.11, 202.12, 202.13, 202.14, 202.15, 202.16, 202.17, 202.18, 202.20, 202.21, 202.28, 202.30, 202.31, 202.32, 202.33, 202.34, 202.36, 202.38, 202.40, 202.42, 202.43, 202.44, 202.47, 202.48, 202.50, 202.51, 202.58, 202.60, 202.62, 202.63,

C22.3, C22.4, C22.7, C22.8, C22.9, C23, C24, C24.0, C24.1, C24.8, C24.9, C25, C25.0, C25.1, C25.2, C25.3, C25.4, C25.7, C25.8, C25.9, C26, C26.0, C26.1, C26.9, C30, C30.0, C30.1, C31, C31.0, C31.1, C31.2, C31.3, C31.8, C31.9, C32, C32.0, C32.1, C32.2, C32.3, C32.8, C32.9, C33, C34, C34.0, C34.00, C34.01, C34.02, C34.1, C34.10, C34.11, C34.12, C34.2, C34.3, C34.30, C34.31, C34.32, C34.8, C34.80, C34.81, C34.82, C34.9, C34.90, C34.91, C34.92, C37, C38, C38.0, C38.1, C38.2, C38.3, C38.4, C38.8, C39, C39.0, C39.9, C40, C40.0, C40.00, C40.01, C40.02, C40.1, C40.10, C40.11, C40.12, C40.2, C40.20, C40.21, C40.22, C40.3, C40.30, C40.31, C40.32, C40.8, C40.80, C40.81, C40.82, C40.9, C40.90, C40.91, C40.92, C41, C41.0, C41.1, C41.2, C41.3, C41.4, C41.9, C43, C43.0, C43.1, C43.10, C43.11, C43.12, C43.2, C43.20, C43.21, C43.22, C43.3, C43.30, C43.31, C43.39, C43.4, C43.5, C43.51, C43.52, C43.59, C43.6, C43.60, C43.61, C43.62, C43.7, C43.70, C43.71, C43.72, C43.8, C43.9, C45, C45.0, C45.1, C45.2, C45.7, C45.9, C46, C46.0, C46.1, C46.2, C46.3, C46.4, C46.5, C46.50, C46.51, C46.52, C46.7, C46.9, C47, C47.0, C47.1, C47.10, C47.11, C47.12, C47.2, C47.20, C47.21, C47.22, C47.3, C47.4, C47.5, C47.6, C47.8, C47.9, C48, C48.0, C48.1, C48.2, C48.8, C49, C49.0, C49.1, C49.10, C49.11, C49.12, C49.2, C49.20, C49.21, C49.22, C49.3, C49.4, C49.5, C49.6, C49.8, C49.9, C49.A, C49.A0, C49.A1, C49.A2, C49.A3, C49.A4, C49.A5, C49.A9, C4A, C4A.0, C4A.1, C4A.10, C4A.11, C4A.12, C4A.2, C4A.20, C4A.21, C4A.22, C4A.3, C4A.30, C4A.31, C4A.39, C4A.4, C4A.5, C4A.51, C4A.52, C4A.59, C4A.6, C4A.60, C4A.61, C4A.62, C4A.7, C4A.70, C4A.71, C4A.72, C4A.8, C4A.9, C50, C50.0, C50.01, C50.011, C50.012, C50.019, C50.02, C50.021, C50.022, C50.029, C50.1, C50.11, C50.111, C50.112, C50.119, C50.12, C50.121, C50.122, C50.129, C50.2, C50.21, C50.211, C50.212, C50.219, C50.22, C50.221, C50.222, C50.229, C50.3, C50.31, C50.311, C50.312, C50.319, C50.32, C50.321, C50.322, C50.329, C50.4, C50.41, C50.411, C50.412, C50.419, C50.42, C50.421, C50.422, C50.429, C50.5, C50.51, C50.511, C50.512, C50.519, C50.52, C50.521, C50.522, C50.529, C50.6, C50.61, C50.611, C50.612, C50.619, C50.62, C50.621, C50.622, C50.629, C50.8, C50.81, C50.811, C50.812, C50.819, C50.82, C50.821, C50.822, C50.829, C50.9, C50.91, C50.911, C50.912, C50.919, C50.92, C50.921, C50.922, C50.929, C51, C51.0, C51.1, C51.2, C51.8, C51.9, C52, C53, C53.0, C53.1, C53.8, C53.9, C54, C54.0, C54.1, C54.2, C54.3, C54.8, C54.9, C55, C56, C56.1, C56.2, C56.9, C57, C57.0, C57.00, C57.01, C57.02, C57.1, C57.10, C57.11, C57.12, C57.2, C57.20, C57.21, C57.22, C57.3, C57.4, C57.7, C57.8, C57.9, C58, C60, C60.0, C60.1, C60.2, C60.8, C60.9, C61, C62, C62.0, C62.00, C62.01, C62.02, C62.1, C62.10, C62.11, C62.12, C62.9, C62.90, C62.91, C62.92, C63, C63.0, C63.00, C63.01, C63.02, C63.1, C63.10, C63.11, C63.12, C63.2, C63.7, C63.8, C63.9, C64, C64.1, C64.2, C64.9, C65, C65.1, C65.2, C65.9, C66, C66.1, C66.2, C66.9, C67, C67.0, C67.1, C67.2, C67.3, C67.4, C67.5, C67.6, C67.7, C67.8, C67.9, C68, C68.0, C68.1, C68.8, C68.9, C69, C69.0, C69.00, C69.01, C69.02, C69.1, C69.10, C69.11, C69.12, C69.2, C69.20, C69.21, C69.22, C69.3, C69.30, C69.31, C69.32, C69.4, C69.40, C69.41, C69.42, C69.5, C69.50, C69.51, C69.52, C69.6, C69.60, C69.61, C69.62, C69.8, C69.80, C69.81, C69.82, C69.9, C69.90, C69.91, C69.92, C73, C74, C74.0, C74.00, C74.01, C74.02, C74.1, C74.10, C74.11, C74.12, C74.9, C74.90, C74.91, C74.92, C75, C75.0, C75.1, C75.2, C75.3, C75.4, C75.5, C75.8, C75.9, C76, C76.0, C76.1, C76.2, C76.3, C76.4, C76.40, C76.41, C76.42, C76.5, C76.50, C76.51, C76.52, C76.8, C77, C77.0, C77.1, C77.2, C77.3, C77.4, C77.5, C77.8, C77.9, C78, C78.0, C78.00, C78.01, C78.02, C78.1, C78.2, C78.3, C78.30, C78.39, C78.4, C78.5, C78.6, C78.7, C78.8, C78.80, C78.89, C79, C79.0, C79.00, C79.01, C79.02, C79.1, C79.10, C79.11, C79.19, C79.2, C79.5, C79.51, C79.52, C79.6, C79.60, C79.61, C79.62, C79.7, C79.70, C79.71, C79.72, C79.8, C79.81, C79.82, C79.89, C79.9, C7A, C7A.0, C7A.00, C7A.01, C7A.010, C7A.011, C7A.012, C7A.019, C7A.02, C7A.020, C7A.021, C7A.022, C7A.023, C7A.024, C7A.025, C7A.026, C7A.029, C7A.09, C7A.090, C7A.091, C7A.092, C7A.093, C7A.094, C7A.095, C7A.096, C7A.098, C7A.1, C7A.8, C7B, C7B.0, C7B.00, C7B.01, C7B.02, C7B.03, C7B.04, C7B.09, C7B.1, C7B.8, C80, C80.0, C80.1, C80.2, C81, C81.0, C81.00, C81.01, C81.02, C81.03, C81.04, C81.05, C81.06, C81.07, C81.08, C81.09, C81.1, C81.10, C81.11, C81.12, C81.13, C81.14, C81.15, C81.16, C81.17, C81.18, C81.19, C81.2, C81.20, C81.21, C81.22, C81.23, C81.24, C81.25, C81.26, C81.27, C81.28, C81.29, C81.3, C81.30, C81.31, C81.32, C81.33, C81.34, C81.35, C81.36, C81.37, C81.38, C81.39, C81.4, C81.40, C81.41, C81.42, C81.43, C81.44, C81.45, C81.46, C81.47, C81.48, C81.49, C81.7, C81.70, C81.71, C81.72, C81.73, C81.74, C81.75, C81.76, C81.77, C81.78, C81.79, C81.9, C81.90, C81.91, C81.92, C81.93, C81.94, C81.95, C81.96, C81.97, C81.98, C81.99, C82, C82.0, C82.00, C82.01, C82.02, C82.03, C82.04, C82.05, C82.06, C82.07, C82.08, C82.09, C82.1, C82.10, C82.11, C82.12, C82.13, C82.14, C82.15, C82.16, C82.17, C82.18, C82.19, C82.2, C82.20, C82.21, C82.22, C82.23, C82.24, C82.25, C82.26, C82.27, C82.28, C82.29, C82.3, C82.30, C82.31, C82.32, C82.33, C82.34, C82.35, C82.36, C82.37, C82.38, C82.39, C82.4, C82.40, C82.41, C82.42, C82.43, C82.44, C82.45, C82.46, C82.47, C82.48, C82.49, C82.5, C82.50, C82.51, C82.52, C82.53, C82.54, C82.55, C82.56, C82.57, C82.58, C82.59,

				202.68, 202.70, 202.72, 202.73, 202.74, 202.75, 202.77, 202.78, 202.8, 202.80, 202.81, 202.82, 202.83, 202.84, 202.85, 202.86, 202.87, 202.88, 202.90, 202.91, 202.92, 202.93, 202.94, 202.95, 202.96, 202.97, 202.98, 203, 203.0, 203.00, 203.01, 203.02, 203.10, 203.11, 203.80, 204.00, 204.01, 204.02, 204.1, 204.10, 204.11, 204.12, 204.80, 204.81, 204.90, 204.91, 204.92, 205.00, 205.01, 205.02, 205.1, 205.10, 205.11, 205.12, 205.30, 205.31, 205.80, 205.81, 205.90, 205.91, 206.00, 206.01, 206.02, 206.10, 206.80, 206.81, 206.90, 207.20, 207.21, 207.80, 207.81, 208.0, 208.00, 208.01, 208.02, 208.1, 208.10, 208.11, 208.20, 208.21, 208.80, 208.81, 208.90, 208.91, 208.92, 209.00, 209.01, 209.02, 209.03, 209.10, 209.11, 209.12, 209.13, 209.16, 209.17, 209.20, 209.21, 209.22, 209.23, 209.24, 209.25, 209.26, 209.27, 209.29, 209.30, 209.31, 209.32, 209.33, 209.34, 209.35, 209.36, 209.40, 209.41, 209.43, 209.50, 209.51, 209.52, 209.53, 209.55, 209.56, 209.57, 209.60, 209.61, 209.63, 209.64, 209.65, 209.66, 209.69, 209.70, 209.71, 209.72, 209.73, 209.74, 209.75, 209.79	C82.6, C82.60, C82.61, C82.62, C82.63, C82.64, C82.65, C82.66, C82.67, C82.68, C82.69, C82.8, C82.80, C82.81, C82.82, C82.83, C82.84, C82.85, C82.86, C82.87, C82.88, C82.89, C82.9, C82.90, C82.91, C82.92, C82.93, C82.94, C82.95, C82.96, C82.97, C82.98, C82.99, C83, C83.0, C83.00, C83.01, C83.02, C83.03, C83.04, C83.05, C83.06, C83.07, C83.08, C83.09, C83.1, C83.10, C83.11, C83.12, C83.13, C83.14, C83.15, C83.16, C83.17, C83.18, C83.19, C83.3, C83.30, C83.31, C83.32, C83.33, C83.34, C83.35, C83.36, C83.37, C83.38, C83.39, C83.5, C83.50, C83.51, C83.52, C83.53, C83.54, C83.55, C83.56, C83.57, C83.58, C83.59, C83.7, C83.70, C83.71, C83.72, C83.73, C83.74, C83.75, C83.76, C83.77, C83.78, C83.79, C83.8, C83.80, C83.81, C83.82, C83.83, C83.84, C83.85, C83.86, C83.87, C83.88, C83.89, C83.9, C83.90, C83.91, C83.92, C83.93, C83.94, C83.95, C83.96, C83.97, C83.98, C83.99, C84, C84.0, C84.00, C84.01, C84.02, C84.03, C84.04, C84.05, C84.06, C84.07, C84.08, C84.09, C84.1, C84.10, C84.11, C84.12, C84.13, C84.14, C84.15, C84.16, C84.17, C84.18, C84.19, C84.4, C84.40, C84.41, C84.42, C84.43, C84.44, C84.45, C84.46, C84.47, C84.48, C84.49, C84.6, C84.60, C84.61, C84.62, C84.63, C84.64, C84.65, C84.66, C84.67, C84.68, C84.69, C84.7, C84.70, C84.71, C84.72, C84.73, C84.74, C84.75, C84.76, C84.77, C84.78, C84.79, C84.9, C84.90, C84.91, C84.92, C84.93, C84.94, C84.95, C84.96, C84.97, C84.98, C84.99, C84.A, C84.A0, C84.A1, C84.A2, C84.A3, C84.A4, C84.A5, C84.A6, C84.A7, C84.A8, C84.A9, C84.Z, C84.Z0, C84.Z1, C84.Z2, C84.Z3, C84.Z4, C84.Z5, C84.Z6, C84.Z7, C84.Z8, C84.Z9, C85, C85.1, C85.10, C85.11, C85.12, C85.13, C85.14, C85.15, C85.16, C85.17, C85.18, C85.19, C85.2, C85.20, C85.21, C85.22, C85.23, C85.24, C85.25, C85.26, C85.27, C85.28, C85.29, C85.8, C85.80, C85.81, C85.82, C85.83, C85.84, C85.85, C85.86, C85.87, C85.88, C85.89, C85.9, C85.90, C85.91, C85.92, C85.93, C85.94, C85.95, C85.96, C85.97, C85.98, C85.99, C88, C88.0, C88.2, C88.3, C88.4, C88.8, C88.9, C90, C90.0, C90.00, C90.01, C90.02, C90.1, C90.10, C90.11, C90.12, C90.2, C90.20, C90.21, C90.22, C90.3, C90.30, C90.31, C90.32, C91, C91.0, C91.00, C91.01, C91.02, C91.1, C91.10, C91.11, C91.12, C91.3, C91.30, C91.31, C91.32, C91.4, C91.40, C91.41, C91.42, C91.5, C91.50, C91.51, C91.52, C91.6, C91.60, C91.61, C91.62, C91.9, C91.90, C91.91, C91.92, C91.A, C91.A0, C91.A1, C91.A2, C91.Z, C91.Z0, C91.Z1, C91.Z2, C92, C92.0, C92.00, C92.01, C92.02, C92.1, C92.10, C92.11, C92.12, C92.2, C92.20, C92.21, C92.22, C92.3, C92.30, C92.31, C92.32, C92.4, C92.40, C92.41, C92.42, C92.5, C92.50, C92.51, C92.52, C92.6, C92.60, C92.61, C92.62, C92.9, C92.90, C92.91, C92.92, C92.A, C92.A0, C92.A1, C92.A2, C92.Z, C92.Z0, C92.Z1, C92.Z2, C93, C93.0, C93.00, C93.01, C93.02, C93.1, C93.10, C93.11, C93.12, C93.3, C93.30, C93.31, C93.32, C93.9, C93.90, C93.91, C93.92, C93.Z, C93.Z0, C93.Z1, C93.Z2, C94, C94.0, C94.00, C94.01, C94.02, C94.2, C94.20, C94.21, C94.22, C94.3, C94.30, C94.31, C94.32, C94.4, C94.40, C94.41, C94.42, C94.6, C94.8, C94.80, C94.81, C94.82, C95, C95.0, C95.00, C95.01, C95.02, C95.1, C95.10, C95.11, C95.12, C95.9, C95.90, C95.91, C95.92, C96, C96.0, C96.2, C96.4, C96.5, C96.6, C96.9, C96.A, C96.Z
**OTHER ARTERIOPATHIES**	Categorical	Levels:<br>0 = Absent<br>1 = Present	Consolidation of the 5 below variables:<br><br>1. Presence of vasculitis at time of index stroke or within 3 months of index stroke<br>2. Presence of fibromuscular dysplasia (FMD) at any time in the EHR<br>3. Presence of reversible cerebrovascular vasoconstriction syndrome (RCVS) at time of index stroke or within 3 months of index stroke<br>4. Presence of moyamoya at any time in the EHR<br>5. Presence of sickle cell disease (SCD) at any time in the EHR	1.<br>437.4, 710.2, 446.4<br><br>2.<br>447.8<br><br>3.<br>435.9<br><br>4.<br>437.5<br><br>5.<br>282.41, 282.42, 282.6, 282.61, 282.62, 282.63, 282.64, 282.68, 289.52	1.<br>I67.7<br><br>2.<br>I77.3<br><br>3.<br>I67.841<br><br>4.<br>I67.5<br><br>5.<br>D57.00, D57.02, D57.1, D57.20, D57.211, D57.212, D57.219, D57.40, D57.419, D57.80, D57.819

**CHRONIC SYSTEMIC DISORDERS**	Categorical	Levels:<br>0 = Absent<br>1 = Present	Consolidation of the 5 below variables:<br><br>1. Presence of chronic liver disease at time of index stroke<br>2. Presence of chronic lung disease at time of index stroke<br>3. Presence of chronic kidney disease (CKD) at time of index stroke<br>4. Presence of end stage renal disease (ESRD) at time of index stroke<br>5. Presence of cirrhosis at time of index stroke or within 3 months of index stroke	1.<br>070.32, 070.33, 070.54, 070.9 , 570, 571.0, 571.2, 571.3, 571.4, 571.40, 571.41, 571.42, 571.49, 571.5, 571.6, 571.8, 571.9, 573.3 , 573.4, 070.22, 070.23, 070.44, 070.6, 456.0, 456.1, 456.2, 456.20, 456.21, 567.23, 572.1, 572.2, 572.3, 572.4, 572.8<br><br>2.<br>490, 491, 491.0, 491.1, 491.2, 491.20, 491.21, 491.22, 491.8, 491.9, 492, 492.0, 492.8, 493, 493.0, 493.00, 493.01, 493.02, 493.1, 493.10, 493.11, 493.12, 493.2, 493.20, 493.21, 493.22, 493.8, 493.81, 493.82, 493.9, 493.90, 493.91, 493.92, 494, 494.0, 494.1, 495, 495.0, 495.1, 495.2, 495.3, 495.4, 495.5, 495.6, 495.7, 495.8, 495.9, 496, 500, 501, 502, 503, 504, 505, 506.4, 508.1<br><br>3.<br>403.00, 403.01, 403.10, 403.11, 403.90, 403.91, 404.00, 404.01, 404.02, 404.03, 404.10, 404.11, 404.12, 404.13, 404.90, 404.91, 404.92, 404.93, 585, 585.1, 585.2, 585.3, 585.4, 585.5, 585.6, 585.9, 586<br><br>4. 585.6<br><br>5. 571.2, 571.5, 571.6	1.<br>B18, B18.0, B18.1, B18.2, B18.8, B18.9, K70.0, K70.1, K70.10, K70.2, K70.3, K70.30, K70.9, K71.3, K71.4, K71.5, K71.7, K73, K73.0, K73.1, K73.2, K73.8, K73.9, K74, K74.0, K74.1, K74.2, K74.3, K74.4, K74.5, K74.6, K74.60, K74.69, K76.0, K76.2, K76.3, K76.4, K76.8, K76.9, Z94.4 I85.0, I86.4, K70.11, K70.31, K70.4, K70.40, K70.41, K71.1, K72.1, K72.9, K76.5, K76.6, K76.7<br><br>2.<br>J40, J41, J41.0, J41.1, J41.8, J42, J43, J43.0, J43.1, J43.2, J43.8, J43.9, J44, J44.0, J44.1, J44.9, J45, J45.2, J45.20, J45.21, J45.22, J45.3, J45.30, J45.31, J45.32, J45.4, J45.40, J45.41, J45.42, J45.5, J45.50, J45.51, J45.52, J45.9, J45.90, J45.901, J45.902, J45.909, J45.99, J45.990, J45.991, J45.998, J47, J47.0, J47.1, J47.9, J60, J61, J62, J62.0, J62.8, J63, J63.0, J63.1, J63.2, J63.3, J63.4, J63.5, J63.6, J64, J65, J66, J66.0, J66.1, J66.2, J66.8, J67, J67.0, J67.1, J67.2, J67.3, J67.4, J67.5, J67.6, J67.7, J67.8, J67.9, J68.4, J70.1, J70.3<br><br>3.<br>I12, I12.0, I12.9, I13, I13.0, I13.1, I13.10, I13.11, I13.2, N18, N18.1, N18.2, N18.3, N18.4, N18.5, N18.6, N18.9, N19, Z49.0, Z94.0, Z99.2<br><br>4. N18.6<br><br>5. K74, K74.0, K74.1, K74.2, K74.3, K74.4, K74.5, K74.6, K74.60, K74.69
**PERIPHERAL VASCULAR DISEASE (PVD)**	Categorical	Levels:<br>0 = Absent<br>1 = Present	Presence of peripheral vascular disease at time of index stroke or within 3 months of index stroke	440, 440.0, 440.1, 440.2, 440.20, 440.21, 440.22, 440.23, 440.24, 440.29, 440.3, 440.30, 440.31, 440.32, 440.4, 440.8, 440.9, 443.0, 443.1, 443.8, 443.81, 443.82, 443.89, 443.9, 447.1, 557, 557.0, 557.1, 557.9	I70, I70.0, I70.1, I70.2, I70.20, I70.201, I70.202, I70.203, I70.208, I70.209, I70.21, I70.211, I70.212, I70.213, I70.218, I70.219, I70.22, I70.221, I70.222, I70.223, I70.228, I70.229, I70.23, I70.231, I70.232, I70.233, I70.234, I70.235, I70.238, I70.239, I70.24, I70.241, I70.242, I70.243, I70.244, I70.245, I70.248, I70.249, I70.25, I70.26, I70.261, I70.262, I70.263, I70.268, I70.269, I70.29, I70.291, I70.292, I70.293, I70.298, I70.299, I70.3, I70.30, I70.301, I70.302, I70.303, I70.308, I70.309, I70.31, I70.311, I70.312, I70.313, I70.318, I70.319, I70.32, I70.321, I70.322, I70.323, I70.328, I70.329, I70.33, I70.331, I70.332, I70.333, I70.334, I70.335, I70.338, I70.339, I70.34, I70.341, I70.342, I70.343, I70.344, I70.345, I70.348, I70.349, I70.35, I70.36, I70.361, I70.362, I70.363, I70.368, I70.369, I70.39, I70.391, I70.392, I70.393, I70.398, I70.399, I70.4, I70.40, I70.401, I70.402, I70.403, I70.408, I70.409, I70.41, I70.411, I70.412, I70.413, I70.418, I70.419, I70.42, I70.421, I70.422, I70.423, I70.428, I70.429, I70.43, I70.431, I70.432, I70.433, I70.434, I70.435, I70.438, I70.439, I70.44, I70.441, I70.442, I70.443, I70.444, I70.445, I70.448, I70.449, I70.45, I70.46, I70.461, I70.462, I70.463, I70.468, I70.469, I70.49, I70.491, I70.492, I70.493, I70.498, I70.499, I70.5, I70.50, I70.501, I70.502, I70.503, I70.508, I70.509, I70.51, I70.511, I70.512, I70.513, I70.518, I70.519, I70.52, I70.521, I70.522, I70.523, I70.528, I70.529, I70.53, I70.531, I70.532, I70.533, I70.534, I70.535, I70.538, I70.539, I70.54, I70.541, I70.542, I70.543, I70.544, I70.545, I70.548, I70.549, I70.55, I70.56, I70.561, I70.562, I70.563, I70.568, I70.569, I70.59, I70.591, I70.592, I70.593, I70.598, I70.599, I70.6, I70.60, I70.601, I70.602, I70.603, I70.608, I70.609, I70.61, I70.611, I70.612, I70.613, I70.618, I70.619, I70.62, I70.621, I70.622, I70.623, I70.628, I70.629, I70.63, I70.631, I70.632, I70.633, I70.634, I70.635, I70.638, I70.639, I70.64, I70.641, I70.642, I70.643, I70.644, I70.645, I70.648, I70.649, I70.65, I70.66, I70.661, I70.662, I70.663, I70.668, I70.669, I70.69, I70.691, I70.692, I70.693, I70.698, I70.699, I70.7, I70.70, I70.701, I70.702, I70.703, I70.708, I70.709, I70.71, I70.711, I70.712, I70.713, I70.718, I70.719, I70.72, I70.721, I70.722, I70.723, I70.728, I70.729, I70.73, I70.731, I70.732, I70.733, I70.734, I70.735, I70.738, I70.739, I70.74, I70.741, I70.742, I70.743, I70.744, I70.745, I70.748, I70.749, I70.75, I70.76, I70.761, I70.762, I70.763, I70.768, I70.769, I70.79, I70.791, I70.792, I70.793, I70.798, I70.799, I70.8, I70.9, I70.90, I70.91, I70.92, I73, I73.0, I73.00, I73.01, I73.1, I73.8, I73.81, I73.89, I73.9, I77.1
**PATENT FORAMEN OVALE (PFO)**	Categorical	Levels:<br>0 = Absent<br>1 = Present	Presence of PFO at any time in the EHR	745.5	Q21.1
**RHEUMATIC DISEASES**	Categorical	Levels:<br>0 = Absent<br>1 = Present	Presence of any rheumatic disease at the time of index stroke or within 3 months of index stroke	710, 710.0, 710.1, 710.2, 710.3, 710.4, 710.5, 710.8, 710.9, 714, 714.0, 714.1, 714.2, 714.4, 714.8, 714.81, 714.89, 714.9, 725	M05, M05.0, M05.00, M05.01, M05.011, M05.012, M05.019, M05.02, M05.021, M05.022, M05.029, M05.03, M05.031, M05.032, M05.039, M05.04, M05.041, M05.042, M05.049, M05.05, M05.051, M05.052, M05.059, M05.06, M05.061, M05.062, M05.069, M05.07, M05.071, M05.072, M05.079, M05.09, M05.1, M05.10, M05.11, M05.111, M05.112, M05.119, M05.12, M05.121, M05.122, M05.129, M05.13,

					M05.131, M05.132, M05.139, M05.14, M05.141, M05.142, M05.149, M05.15, M05.151, M05.152, M05.159, M05.16, M05.161, M05.162, M05.169, M05.17, M05.171, M05.172, M05.179, M05.19, M05.2, M05.20, M05.21, M05.211, M05.212, M05.219, M05.22, M05.221, M05.222, M05.229, M05.23, M05.231, M05.232, M05.239, M05.24, M05.241, M05.242, M05.249, M05.25, M05.251, M05.252, M05.259, M05.26, M05.261, M05.262, M05.269, M05.27, M05.271, M05.272, M05.279, M05.29, M05.3, M05.30, M05.31, M05.311, M05.312, M05.319, M05.32, M05.321, M05.322, M05.329, M05.33, M05.331, M05.332, M05.339, M05.34, M05.341, M05.342, M05.349, M05.35, M05.351, M05.352, M05.359, M05.36, M05.361, M05.362, M05.369, M05.37, M05.371, M05.372, M05.379, M05.39, M05.4, M05.40, M05.41, M05.411, M05.412, M05.419, M05.42, M05.421, M05.422, M05.429, M05.43, M05.431, M05.432, M05.439, M05.44, M05.441, M05.442, M05.449, M05.45, M05.451, M05.452, M05.459, M05.46, M05.461, M05.462, M05.469, M05.47, M05.471, M05.472, M05.479, M05.49, M05.5, M05.50, M05.51, M05.511, M05.512, M05.519, M05.52, M05.521, M05.522, M05.529, M05.53, M05.531, M05.532, M05.539, M05.54, M05.541, M05.542, M05.549, M05.55, M05.551, M05.552, M05.559, M05.56, M05.561, M05.562, M05.569, M05.57, M05.571, M05.572, M05.579, M05.59, M05.6, M05.60, M05.61, M05.611, M05.612, M05.619, M05.62, M05.621, M05.622, M05.629, M05.63, M05.631, M05.632, M05.639, M05.64, M05.641, M05.642, M05.649, M05.65, M05.651, M05.652, M05.659, M05.66, M05.661, M05.662, M05.669, M05.67, M05.671, M05.672, M05.679, M05.69, M05.7, M05.70, M05.71, M05.711, M05.712, M05.719, M05.72, M05.721, M05.722, M05.729, M05.73, M05.731, M05.732, M05.739, M05.74, M05.741, M05.742, M05.749, M05.75, M05.751, M05.752, M05.759, M05.76, M05.761, M05.762, M05.769, M05.77, M05.771, M05.772, M05.779, M05.79, M05.8, M05.80, M05.81, M05.811, M05.812, M05.819, M05.82, M05.821, M05.822, M05.829, M05.83, M05.831, M05.832, M05.839, M05.84, M05.841, M05.842, M05.849, M05.85, M05.851, M05.852, M05.859, M05.86, M05.861, M05.862, M05.869, M05.87, M05.871, M05.872, M05.879, M05.89, M05.9, M06, M06.0, M06.00, M06.01, M06.011, M06.012, M06.019, M06.02, M06.021, M06.022, M06.029, M06.03, M06.031, M06.032, M06.039, M06.04, M06.041, M06.042, M06.049, M06.05, M06.051, M06.052, M06.059, M06.06, M06.061, M06.062, M06.069, M06.07, M06.071, M06.072, M06.079, M06.08, M06.09, M06.1, M06.2, M06.20, M06.21, M06.211, M06.212, M06.219, M06.22, M06.221, M06.222, M06.229, M06.23, M06.231, M06.232, M06.239, M06.24, M06.241, M06.242, M06.249, M06.25, M06.251, M06.252, M06.259, M06.26, M06.261, M06.262, M06.269, M06.27, M06.271, M06.272, M06.279, M06.28, M06.29, M06.3, M06.30, M06.31, M06.311, M06.312, M06.319, M06.32, M06.321, M06.322, M06.329, M06.33, M06.331, M06.332, M06.339, M06.34, M06.341, M06.342, M06.349, M06.35, M06.351, M06.352, M06.359, M06.36, M06.361, M06.362, M06.369, M06.37, M06.371, M06.372, M06.379, M06.38, M06.39, M06.4, M06.8, M06.80, M06.81, M06.811, M06.812, M06.819, M06.82, M06.821, M06.822, M06.829, M06.83, M06.831, M06.832, M06.839, M06.84, M06.841, M06.842, M06.849, M06.85, M06.851, M06.852, M06.859, M06.86, M06.861, M06.862, M06.869, M06.87, M06.871, M06.872, M06.879, M06.88, M06.89, M06.9, M31.5, M32, M32.0, M32.1, M32.10, M32.11, M32.12, M32.13, M32.14, M32.15, M32.19, M32.8, M32.9, M33, M33.0, M33.00, M33.01, M33.02, M33.03, M33.09, M33.1, M33.10, M33.11, M33.12, M33.13, M33.19, M33.2, M33.20, M33.21, M33.22, M33.29, M33.9, M33.90, M33.91, M33.92, M33.93, M33.99, M34, M34.0, M34.1, M34.2, M34.8, M34.81, M34.82, M34.83, M34.89, M34.9, M35.1, M35.3, M36.0
**SMOKING STATUS**	Categorical	Levels:<br>0 = nonsmoker<br>1 = smoking	Smoking status at time of index stroke	n/a	n/a
**GIANT CELL TEMPORAL ARTERITIS (GCA)**	Categorical	Levels:<br>0 = Absent<br>1 = Present	Presence of any GCA at the time of index stroke or within 3 months of index stroke		

Table S4. List of variables and the respective counts and percentages in different female groups based on the different age brackets. ** p<0.05 between cases and control for the age bracket.*

	CASE GROUP										CONTROL GROUP									
	≤40	≤45	≤50	≤55	≤60	≤65	≤70	≤75	<80	ALL	≤40	≤45	≤50	≤55	≤60	≤65	≤70	≤75	<80	ALL
N	88	155	269	433	710	1013	1396	1856	2369	3663	1988	2577	3325	4204	5211	6206	7350	8645	9938	14670
Arteriopathies, N (%)	15 (17) *	28 (18.1) *	43 (16) *	73 (16.9) *	102 (14.4) *	146 (14.4) *	191 (13.7) *	258 (13.9) *	352 (14.9) *	545 (14.9) *	11 (0.6) *	18 (0.7) *	22 (0.7) *	36 (0.9) *	43 (0.8) *	57 (0.9) *	78 (1.1) *	99 (1.1) *	115 (1.2) *	188 (1.3) *
Chronic Systemic Disorder, N (%)	22 (25.0) *	36 (23.2) *	73 (27.1) *	127 (29.3) *	230 (32.4) *	323 (31.9) *	461 (33.0) *	616 (33.2) *	804 (33.9) *	1332 (36.4) *	287 (14.4) *	411 (15.9) *	594 (17.9) *	834 (19.8) *	1121 (21.5) *	1426 (23.0) *	1790 (24.4) *	2178 (25.2) *	2513 (25.3) *	3534 (24.1) *
Hypercoagulable State, N (%)	11 (12.5) *	17 (11.0) *	24 (8.9) *	31 (7.2) *	41 (5.8) *	51 (4.1) *	57 (4.1) *	63 (3.4) *	65 (2.7) *	79 (2.2) *	22 (1.1) *	33 (1.3) *	46 (1.4) *	59 (1.4) *	71 (1.4) *	82 (1.3) *	102 (1.4) *	116 (1.3) *	123 (1.2) *	150 (1.0) *
Atrial Fibrillation or Flutter, N (%)	3 (3.4)	6 (3.9) *	8 (3.0) *	14 (3.2) *	35 (4.9) *	77 (7.6) *	151 (10.8) *	249 (13.4) *	382 (16.1) *	881 (24.1) *	16 (0.8)	20 (0.8) *	41 (1.2) *	66 (1.6) *	126 (2.4) *	223 (3.6) *	377 (5.1) *	646 (7.5) *	993 (10.0) *	2499 (17.0) *
Alcohol Dependence or Abuse, N (%)	1 (1.1)	2 (1.3)	6 (2.2)	17 (3.9)	30 (4.2)	41 (4.0)	55 (3.9)	60 (3.2)	71 (3.0)	80 (2.2)	56 (2.8)	93 (3.6)	138 (4.2)	193 (4.6)	237 (4.5)	267 (4.3)	279 (3.8)	295 (3.4)	305 (3.1)	313 (2.1)
Congestive Heart Failure, N (%)	4 (4.5) *	5 (3.2) *	9 (3.3) *	14 (3.2)	40 (5.6) *	69 (6.8) *	110 (7.9) *	169 (9.1)	227 (9.6)	495 (13.5)	10 (0.5) *	26 (1.0) *	49 (1.5) *	110 (2.6)	197 (3.8) *	315 (5.1) *	466 (6.3) *	695 (8.0)	925 (9.3)	1909 (13.0)
Diabetes Mellitus, N (%)	10 (11.4) *	21 (13.5) *	49 (18.2) *	97 (22.4) *	189 (26.6) *	308 (30.4) *	451 (32.3) *	626 (33.7) *	790 (33.3) *	1128 (30.8) *	113 (5.7) *	185 (7.2) *	277 (8.3) *	425 (10.1) *	655 (12.6) *	908 (14.6) *	1221 (16.6) *	1599 (18.5) *	1974 (19.9) *	3006 (20.5) *
Drug Dependence or Abuse, N (%)	9 (10.2) *	13 (8.4) *	18 (6.7)	23 (5.3)	32 (4.5)	33 (3.3)	40 (2.9)	43 (2.3)	49 (2.1)	51 (1.4)	84 (4.2) *	116 (4.5) *	145 (4.4)	168 (4.0)	194 (3.7)	210 (3.4)	221 (3.0)	224 (2.6)	227 (2.3)	231 (1.6)
Family History of Stroke or MI, N (%)	29 (33.0) *	53 (34.2) *	107 (39.8) *	181 (41.8) *	303 (42.7) *	461 (45.5) *	647 (46.3) *	845 (45.5) *	1082 (45.7) *	1573 (42.9) *	382 (19.2) *	554 (21.5) *	805 (24.2) *	1158 (27.5) *	1566 (30.1) *	1982 (31.9) *	2461 (33.5) *	2992 (34.6) *	3505 (35.3) *	4975 (33.9) *
History of Myocardial Infarction, N (%)	5 (5.7) *	6 (3.9) *	9 (3.3) *	21 (4.8) *	44 (6.2) *	67 (6.6) *	101 (7.2) *	138 (7.4) *	179 (7.6) *	311 (8.5) *	7 (0.4) *	13 (0.5) *	27 (0.8) *	50 (1.2) *	82 (1.6) *	121 (1.9) *	183 (2.5) *	275 (3.2) *	343 (3.5) *	634 (4.3) *
Peripheral Vascular Disease, N (%)	0 (0.0)	3 (1.9) *	9 (3.3) *	25 (5.8) *	64 (9.0) *	100 (9.9) *	150 (10.7) *	214 (11.5) *	282 (11.9) *	490 (13.4) *	4 (0.2)	7 (0.3) *	20 (0.6) *	36 (0.9) *	66 (1.3) *	86 (1.4) *	130 (1.8) *	200 (2.3) *	264 (2.7) *	526 (3.6) *
Patent Foramen Ovale, N (%)	24 (27.3) *	39 (25.2) *	62 (23.0) *	88 (20.3) *	116 (16.3) *	148 (14.6) *	175 (12.5) *	199 (10.7) *	223 (9.4) *	258 (7.0) *	14 (0.7) *	20 (0.8) *	26 (0.8) *	30 (0.7) *	39 (0.7) *	48 (0.8) *	64 (0.9) *	73 (0.8) *	86 (0.9) *	102 (0.7) *
Current Smoking, N (%)	23 (26.1)	46 (29.7)	79 (29.4)	127 (29.3)	206 (29.0)	273 (26.9)	350 (25.1)	395 (21.3)	428 (18.1) *	453 (12.4) *	596 (30.0)	785 (30.5)	1025 (30.8)	1275 (30.3)	1537 (29.5)	1714 (27.6)	1860 (25.3)	1970 (22.8)	2055 (20.7) *	2145 (14.6) *
Body Mass Index Elevated (>25) , N (%)	69 (78.4) *	121 (78.1) *	214 (79.6) *	340 (78.5) *	547 (77.0) *	785 (77.5) *	1082 (77.5) *	1433 (77.2) *	1801 (76.0) *	2508 (68.5) *	1139 (57.3) *	1531 (59.4) *	2017 (60.7) *	2603 (61.9) *	3304 (64.4) *	4022 (64.8) *	4828 (65.7) *	5733 (66.3) *	6593 (66.3) *	8983 (61.2) *
Arterial Dissection, N (%)	2 (2.3) *	3 (1.9) *	5 (1.9) *	5 (1.2) *	5 (0.7) *	5 (0.5) *	6 (0.4) *	6 (0.3) *	7 (0.3) *	9 (0.2) *	1 (0.1) *	2 (0.1) *	2 (0.1) *	2 (0.0) *	2 (0.0) *	2 (0.0) *	2 (0.0) *	2 (0.0) *	2 (0.0) *	2 (0.0) *
Neoplasm, N (%)	1 (1.1)	5 (3.2)	11 (4.1)	22 (5.1)	50 (7.0)	77 (7.6)	135 (9.7)	207 (11.2)	285 (12.0)	533 (14.6)	37 (1.9)	67 (2.6)	127 (3.8)	218 (5.2)	360 (6.9)	526 (8.5)	735 (10.0)	971 (11.2)	1208 (12.2)	1957 (13.3)
Rheumatic Disease, N (%)	3 (3.4)	8 (5.2)	11 (4.1)	20 (4.6)	38 (5.4)	61 (6.0)	96 (6.9)	127 (6.8)	174 (7.3)	263 (7.2)	53 (2.7)	80 (3.1)	123 (3.7)	175 (4.2)	238 (4.6)	326 (5.3)	408 (5.6)	529 (6.1)	643 (6.5)	1027 (7.0)

	≤40	≤45	≤50	≤55	≤60	≤65	≤70	≤75	<80	ALL	≤40	≤45	≤50	≤55	≤60	≤65	≤70	≤75	<80	ALL
Migraines, N (%)	26 (29.5) *	44 (28.5) *	68 (25.3) *	93 (21.5) *	122 (17.2) *	142 (14.0) *	163 (11.7) *	184 (9.9) *	205 (9.7) *	239 (6.5) *	193 (9.7) *	273 (10.6) *	368 (11.1) *	464 (11.0) *	523 (10.0) *	588 (9.5) *	638 (8.7) *	674 (7.8) *	696 (7.0) *	740 (5.0) *
Hypertension, N (%)	24 (7.3) *	49 (31.6) *	116 (43.1) *	227 (52.4) *	419 (59.0) *	649 (64.1) *	966 (69.2) *	1338 (72.1) *	1764 (74.5) *	2862 (78.1) *	269 (13.6) *	436 (16.9) *	721 (21.7) *	1142 (27.2) *	1699 (32.6) *	2333 (37.6) *	3154 (42.9) *	4145 (47.9) *	5168 (52.0) *	9035 (61.6) *
Dyslipidemia, N (%)	12 (13.6) *	27 (17.4) *	79 (29.4) *	163 (37.6) *	312 (43.9) *	497 (49.1) *	755 (54.1) *	1058 (57.0) *	1390 (58.7) *	2206 (60.2) *	100 (5.0) *	197 (7.6) *	365 (11.0) *	607 (14.4) *	950 (18.2) *	1342 (21.6) *	1853 (25.2) *	2484 (28.7) *	3117 (31.4) *	5190 (35.4) *
Mood Disorder, N (%)	21 (23.9)	31 (20.0)	68 (25.3)	122 (28.2)	201 (28.3)	275 (27.1)	371 (26.6)	464 (25.0)	549 (23.2)	766 (20.9) *	389 (19.6)	551 (21.4)	780 (23.5)	1038 (24.7)	1314 (25.2)	1588 (25.6)	1841 (25.0)	2079 (24.0)	2284 (23.0)	2785 (19.0) *
Anxiety Disorder, N (%)	24 (27.3) *	39 (25.2) *	65 (25.2) *	106 (24.5) *	167 (23.5) *	219 (21.6) *	295 (21.1)	375 (20.2)	481 (20.3) *	720 (19.7) *	289 (14.5) *	414 (16.1) *	570 (17.1) *	762 (18.1) *	977 (18.7) *	1165 (18.8) *	1388 (18.9)	1620 (18.7)	1815 (18.3) *	2401 (16.4) *
Giant Cell Temporal Arteritis, N (%)	0 (0.0)	0 (0.0)	0 (0.0)	0 (0.0)	0 (0.0)	0 (0.0)	0 (0.0)	1 (0.1)	2 (0.1)	10 (0.03)	0 (0.0)	0 (0.0)	0 (0.0)	1 (0.1)	3 (0.1)	5 (0.1)	10 (0.1)	18 (0.2)	25 (0.3)	63 (0.4)

Table S5. List of variables and the respective counts and percentages in different male groups based on the different age brackets. ** p<0.05 between cases and control for the age bracket.*

	CASE GROUP										CONTROL GROUP									
	**≤40**	**≤45**	**≤50**	**≤55**	**≤60**	**≤65**	**≤70**	**≤75**	<80	**ALL**	**≤40**	**≤45**	**≤50**	**≤55**	**≤60**	**≤65**	**≤70**	**≤75**	<80	ALL
N	80	179	348	613	1052	1534	2084	2582	3066	3892	3572	4410	5506	6867	8272	9612	11023	12330	13578	16397
Arteriopathies, N (%)	9 (11.2) *	20 (11.2) *	31 (8.9) *	61 (10.0) *	103 (9.8) *	149 (9.7) *	203 (9.7) *	263 (10.2) *	325 (10.6) *	442 (11.4) *	8 (0.2) *	10 (0.2) *	14 (0.3) *	18 (0.3) *	24 (0.3) *	28 (0.3) *	40 (0.4) *	54 (0.4) *	66 (0.5) *	84 (0.5) *
Chronic Systemic Disorder, N (%)	20 (25.0) *	35 (19.6) *	68 (19.5) *	118 (19.2) *	240 (22.8) *	384 (25.0) *	543 (26.1) *	706 (27.3) *	910 (29.7) *	1280 (32.9) *	255 (7.1) *	327 (7.4) *	477 (8.7) *	762 (11.1) *	1070 (12.9) *	1388 (14.4) *	1805 (16.4) *	2180 (17.7) *	2560 (18.9) *	3367 (20.5) *
Hypercoagulable State, N (%)	6 (7.5) *	11 (6.1) *	24 (6.9) *	26 (4.2) *	38 (3.6) *	48 (3.1) *	57 (2.7) *	63 (2.4) *	66 (2.2) *	70 (1.8) *	10 (0.3) *	14 (0.3) *	25 (0.5) *	36 (0.5) *	52 (0.6) *	62 (0.6) *	78 (0.7) *	85 (0.7) *	97 (0.7) *	114 (0.7) *
Atrial Fibrillation or Flutter, N (%)	1 (1.2)	3 (1.7)	12 (3.4)	33 (5.4) *	82 (7.8) *	144 (9.4) *	250 (12.0) *	381 (14.8) *	537 (17.5) *	840 (21.6) *	35 (1.0)	58 (1.3)	100 (1.8)	172 (2.5) *	288 (3.5) *	445 (4.6) *	724 (6.6) *	1062 (8.6) *	1453 (10.7) *	2566 (15.6) *
Alcohol Dependence or Abuse, N (%)	4 (5.0)	16 (8.9) *	38 (10.9) *	70 (11.4) *	112 (10.6) *	156 (10.2) *	197 (9.5) *	229 (8.9) *	246 (8.0) *	261 (6.7) *	106 (3.0)	160 (3.6) *	250 (4.5) *	377 (5.5) *	508 (6.1) *	595 (6.2) *	656 (6.0) *	708 (5.7) *	736 (5.4) *	771 (4.7) *
Congestive Heart Failure, N (%)	4 (5.0) *	7 (3.9) *	18 (5.2) *	33 (5.4) *	67 (6.4) *	107 (7.0) *	164 (7.9) *	216 (8.4) *	284 (9.3)	452 (11.6)	25 (0.7) *	43 (1.0) *	86 (1.6) *	157 (2.3) *	240 (2.9) *	382 (4.0) *	600 (5.4) *	845 (6.9) *	1132 (8.3)	1841 (11.2)
Diabetes Mellitus, N (%)	14 (17.5) *	35 (19.6) *	78 (22.4) *	159 (25.9) *	291 (27.7) *	439 (28.6) *	653 (31.3) *	825 (32.0) *	996 (32.5) *	1254 (32.2) *	82 (2.3) *	143 (3.2) *	258 (4.8) *	464 (6.8) *	725 (8.8) *	1037 (10.8) *	1443 (13.1) *	1857 (15.1) *	2248 (16.6) *	3017 (18.4) *
Drug Dependence or Abuse, N (%)	6 (7.5)	11 (6.1)	20 (5.7) *	25 (4.1)	39 (3.7)	45 (2.9)	52 (2.5)	57 (2.2)	58 (1.9)	58 (1.5)	116 (3.2)	142 (3.2)	170 (3.1) *	198 (2.9)	229 (2.8)	252 (2.6)	264 (2.4)	269 (2.2)	272 (2.0)	273 (1.7)
Family History of Stroke or MI, N (%)	21 (26.2) *	50 (27.9) *	111 (31.9) *	232 (37.8) *	401 (38.1) *	595 (38.8) *	847 (40.6) *	1063 (41.2) *	1263 (41.2) *	1543 (39.6) *	300 (8.4) *	476 (10.8) *	735 (13.3) *	1118 (16.3) *	1528 (18.5) *	1945 (20.2) *	2424 (22.0) *	2867 (23.3) *	3300 (24.3) *	4097 (25.0) *
History of Myocardial Infarction, N (%)	3 (3.8) *	9 (5.0) *	22 (6.3) *	48 (7.8) *	92 (8.7) *	138 (9.0) *	220 (10.6) *	284 (11.0) *	362 (11.8) *	498 (12.8) *	11 (0.3) *	24 (0.5) *	54 (1.0) *	100 (1.5) *	144 (1.7) *	226 (2.4) *	337 (3.1) *	449 (3.6) *	573 (4.2) *	794 (4.8) *
Peripheral Vascular Disease, N (%)	1 (1.2)	7 (3.9)	23 (6.6)	40 (6.5)	101 (9.6)	163 (10.6)	265 (12.7)	360 (13.9)	474 (15.5)	655 (16.8)	0 (0.0)	3 (0.1)	8 (0.1)	35 (0.5)	56 (0.7)	117 (1.2)	195 (1.8)	291 (2.4)	407 (3.0)	637 (3.9)

	*	*	*	*	*	*	*	*	*	*	*	*	*	*	*	*	*	*	*	*
Patent Foramen Ovale, N (%)	22 (27.5) *	40 (22.3) *	67 (19.3) *	103 (16.8) *	154 (14.6) *	193 (12.6) *	235 (11.3) *	267 (10.3) *	300 (9.8) *	328 (8.4) *	10 (0.3) *	12 (0.3) *	19 (0.3) *	25 (0.4) *	33 (0.4) *	47 (0.5) *	55 (0.5) *	60 (0.5) *	64 (0.5) *	78 (0.5) *
Current Smoking, N (%)	18 (22.5)	41 (22.9)	84 (24.1)	159 (25.9)	276 (26.2)	380 (24.8)	480 (23.0)	543 (21.0)	585 (19.1)	629 (16.2)	809 (22.6)	1015 (23.0)	1292 (23.5)	1658 (24.1)	2006 (24.3)	2281 (23.7)	2470 (22.4)	2631 (21.3)	2740 (20.2)	2829 (17.3)
Body Mass Index Elevated (>25), N (%)	63 (78.8) *	144 (80.4) *	278 (79.9) *	497 (81.1) *	836 (79.5) *	1219 (79.5) *	1661 (79.7) *	2055 (79.6) *	2420 (78.9) *	2956 (76.0) *	2064 (57.8) *	2667 (60.5) *	3447 (62.6) *	4473 (65.1) *	5484 (66.3) *	6431 (66.9) *	7447 (67.6) *	8364 (67.8) *	9217 (67.9) *	10720 (65.4) *
Arterial Dissection, N (%)	1 (1.2)	3 (1.7) *	3 (0.9) *	5 (0.8) *	8 (0.8) *	8 (0.5) *	9 (0.4) *	10 (0.4) *	10 (0.3) *	11 (0.3) *	5 (0.1)	6 (0.1) *	6 (0.1) *	6 (0.1) *	6 (0.1) *	7 (0.1) *	7 (0.1) *	8 (0.1) *	8 (0.1) *	9 (0.1) *
Neoplasm, N (%)	3 (3.8)	6 (3.4)	10 (2.9)	19 (3.1)	59 (5.6) *	98 (6.4)	170 (8.2)	272 (10.5) *	371 (12.1) *	617 (15.9) *	34 (1.0)	60 (1.4)	113 (2.1)	194 (2.8)	325 (3.9) *	518 (5.4)	764 (6.9)	1004 (8.1) *	1298 (9.6) *	2050 (12.5) *
Rheumatic Disease, N (%)	0 (0.0)	2 (1.1)	4 (1.1)	9 (1.5) *	18 (1.7) *	25 (1.6)	35 (1.7)	49 (1.9)	61 (2.0)	94 (2.4)	16 (0.4)	18 (0.4)	29 (0.5)	44 (0.6) *	77 (0.9) *	111 (1.2)	147 (1.3)	196 (1.5)	239 (1.8)	352 (2.1)
Migraines, N (%)	4 (5.0)	13 (7.3) *	24 (6.9) *	36 (5.9) *	53 (5.0) *	64 (4.2) *	82 (3.9) *	87 (3.4) *	94 (3.1) *	102 (2.6) *	68 (0.4)	88 (2.0) *	114 (2.1) *	137 (2.0) *	164 (2.0) *	188 (2.0) *	198 (1.8) *	211 (1.7) *	220 (1.6) *	225 (1.4) *
Hypertension, N (%)	37 (46.2) *	93 (52.0) *	208 (59.8) *	405 (66.1) *	755 (71.8) *	1120 (73.0) *	1571 (75.4) *	1991 (77.1) *	2405 (78.4) *	3082 (79.2) *	367 (10.3) *	644 (14.6) *	1071 (19.5) *	1743 (24.4) *	2524 (30.5) *	3376 (35.1) *	4371 (39.7) *	5328 (43.2) *	6262 (46.1) *	8440 (51.5) *
Dyslipidemia, N (%)	26 (32.5) *	69 (38.5) *	153 (44.0) *	304 (49.6) *	574 (54.6) *	869 (56.6) *	1255 (60.2) *	1570 (60.8) *	1894 (61.8) *	2414 (62.0) *	97 (2.7) *	199 (4.5) *	394 (7.2) *	717 (10.4) *	1133 (13.7) *	1607 (16.7) *	2224 (20.2) *	2818 (22.9) *	2444 (25.4) *	4826 (29.4) *
Mood Disorder, N (%)	12 (15.0) *	28 (15.6) *	52 (14.9) *	81 (13.2) *	148 (14.1) *	223 (14.5) *	299 (14.3) *	355 (13.7) *	427 (13.9) *	510 (13.1) *	256 (7.2) *	353 (8.0) *	491 (8.9) *	675 (9.8) *	883 (10.7) *	1070 (11.1) *	1257 (11.4) *	1393 (11.3) *	1539 (11.3) *	1764 (10.8) *
Anxiety Disorder, N (%)	8 (10.0)	22 (12.3) *	44 (12.6) *	68 (11.1) *	121 (11.5) *	169 (11.0) *	236 (11.3) *	278 (10.8) *	318 (10.4) *	391 (10.0) *	181 (5.1)	263 (6.0) *	363 (6.6) *	511 (7.4) *	647 (7.8) *	772 (8.0) *	916 (8.3) *	1036 (8.4) *	1147 (8.4) *	1319 (8.0) *
Giant Cell Temporal Arteritis, N (%)	0 (0.0)	0 (0.0)	0 (0.0)	0 (0.0)	0 (0.0)	0 (0.0)	1 (0.0)	2 (0.1)	2 (0.1)	5 (0.1)	0 (0.0)	0 (0.0)	1 (0.0)	1 (0.0)	2 (0.0)	5 (0.1)	7 (0.1)	7 (0.1)	12 (0.1)	26 (0.2)

Table S6. The best model for each age bracket for males and females, as well as optimized model parameters.

Age Group	Model (model parameters)
Female	
ALL	GLM
<40	GLM
<45	RF (mtry=6)
<50	GLM
<55	GLM
<60	GLM
<65	GLM
<70	GLM
<75	XGB (Max depth=8 eta=0.52; Rate drop=0.19; Skip drop=0.35; Min child weight=1; subsample=0.85; Colsamplebytree=0.60; gamma=6.76; nrounds=67)
Male	
ALL	GLM
<40	GLM
<45	RF (mtry=3)
<50	GLM
<55	GLM
<60	GLM
<65	GLM
<70	GLM
<75	GLM

Table S7. Studies on young stroke patients with the respective findings and the age cut-point used.

Abbreviations include female (F), male (M), oral contraceptive pill (OCP), history (Hx), hypertension (HTN), odds ratio (OR), diabetes mellitus (DM), congestive heart failure (CHF), International Pediatric Stroke Study (IPSS) Criteria, Trial of Org 10172 in Acute Stroke Treatment (TOAST) Criteria, peripheral arterial disease (PAD), atrial fibrillation (AF), ischemic heart disease (IHD), risk factor (RF), genome wide association study (GWAS).

Study	Type	Age	Sample	Findings
(2019)	Chart review	50	5257 first-ever stroke from Dutch Hospital Registry; 55.3% ischemic stroke	• Incidence of all types of "young stroke" increased from 1998 to 2010, driven by an increase in those aged over 35 years.<br>• F>M in all age cohorts may be explained by higher rates of migraine, coagulopathy, OCPs and pregnancy in females.
Di Giuli et al. (2017)	Cohort	45	2485 first-ever ischemic stroke with a Hx of migraine	• Migraine without aura independently associated with cervical dissection stroke etiology in young
Aigner et al. (2017)	Case Control	55	2125 first time strokes from the "Stroke in Young Fabry Patients" registry (2007-2010) vs. 8500 controls from "GEDA" study (2009-2010)	• Low physical activity and HTN were the most important factors in determining risk of stroke.<br>• HTN, low activity, hyperlipidemia, DM, CHF, smoking, binge drinking, and obesity together accounted for 78.9% of strokes in young.
Li et al. (2017)	Retrospective cohort	45	1,395 first time ischemic stroke	• Vascular disease, especially premature atherosclerosis, is the most significant risk factor for ischemic stroke in the young adult population of northern China
Cheng et al. (2016)	Cast Control	49	1090 cases and 1154 controls	• In adjusted analysis (controlling for alcohol, smoking and HTN) the OR for stroke secondary to acute cocaine use by any route was 5.7 (95%CI, 1.7-19.7) indicating a causal association between acute cocaine use and risk of young stroke.
van Alebeek et al. (2017)	Prospective cohort	50	656 Patients enrolled in FUTURE study who had ischemic or hemorrhagic stroke, or a TIA	• Stroke etiology classified according to IPSS and TOAST criteria - 226 patients had stroke of unknown etiology based on TOAST, but 188 of these were assigned an etiology based on IPSS criteria.<br>• IPSS criteria (for pediatric strokes) includes categories of arteriopathy, cardiac disorders, chronic systemic conditions, prothrombotic states, acute systemic conditions, chronic head and neck disorders, acute head and neck disorders, infection and risk factors for atherosclerosis in adulthood (+ added pregnancy / postpartum category for females).
Rohr et al. (1996)	Case Control	44	296 cases of first-time ischemic stroke and 1220 controls	• HTN, DM, and current smoking are the most important risk factors in young adults
Ji et al. (2013)	Retrospective cohort	45	150 ischemic stroke/TIA patients from the AHA "Get with The Guidelines" database	• 47% had cardioembolic etiology<br>• Multiple infarcts (31%) occurred more in <35-year-olds<br>• Young patient risk factor profile included 20% with HTN, 10% with DM, 38% with dyslipidemia, 34% smokers.

				• Young adults have heterogeneous causes for stroke, but traditional vascular risk factors are very prevalent.
Renna et al. (2014)	Prospective cohort	50	150 patients admitted with ischemic stroke.	• Etiologies classified by TOAST.<br>• 53% had dyslipidemia, 47% had smoking, 39% had HTN, 33% had PFO, 30% had cardioembolic 11% had large vessel disease, 8% had small vessel disease, 44% had stroke of unknown etiology.<br>• Traditional risk factors are common among young adults with stroke.
Kristensen et al. (1997)	Retrospective cohort	44	Part 1: 88 patients from MONICA cohort<br>Part 2: referred to university hospital with ischemic stroke (107 patients)	• Classified by modified TOAST criteria<br>• Spontaneous cervical arterial dissection was the leading probable etiology (13%).<br>• There was a higher than predicted incidence of stroke in young that was not explained by premature atherosclerosis.
Musolino et al. (2003)	Prospective cohort	45	60 patients with stroke (n=55) or TIA (n=5) followed up for 1 or more years for recurrence and post stroke disability.	• Atherothrombotic causes were more common >35-year-olds<br>• Migraine was more common in females and smoking was more common in males<br>• 61% had two or more RF present
Putaala et al. (2012)	Cross-sectional cohort	49	First ever ischemic stroke patients from prospective or consecutive stroke registries in Europe.	• The most common RF were smoking, HTN and dyslipidemia<br>• Males were older and more commonly had dyslipidemia, coronary heart disease and were smokers compared to females<br>• Fam Hx of stroke, PAD, AF, smoking, HTN, DM, IHD and dyslipidemia were positive correlated with age in both genders (i.e. became increasingly prevalent as patients aged)
Bejot et al. (2013)	Prospective cohort	55	First ever ischemic stroke patients from Dijon Registry	• Incidence of stroke in people under 55 increased over 1985-2011.<br>• Smoking was the most common RF in young patients.
Jaworek et al. (2021)	Meta analysis	59	Analysis of GWAS study data from various studies across North America, Europe and Asia looking at early onset ischemic stroke patients (16 927) versus controls (576 353).	• A relationship between genetic variants at the known stroke locus *ABO* and venous thrombosis were identified, pointing to a role of prothrombotic factors in early onset stroke compared to later onset stroke.

Table S8. Model AUROC and Accuracy for all the models.

		**FEMALE**			**MALE**	
CRITERIA	MODEL	**AUROC**	**ACCURACY**		**AUROC**	**ACCURACY**
≤ 40	GLM	0.803461753	0.96875		0.828431373	0.979452055
≤ 40	RF	0.829006142	0.961538462		0.896095938	0.980821918
≤ 40	SVM	0.841359576	0.956730769		0.864058123	0.976712329
≤ 40	XGB	0.831728085	0.961538462		0.923231793	0.978082192
≤ 45	GLM	0.872972127	0.956043956		0.927642353	0.968409586
≤ 45	RF	0.873222675	0.954212454		0.898163895	0.968409586
≤ 45	SVM	0.82793611	0.952380952		0.861126858	0.953159041
≤ 45	XGB	0.886783589	0.956043956		0.920808138	0.965141612
≤ 46	GLM	0.834217437	0.949913644		0.938984624	0.970863684
≤ 46	RF	0.954516807	0.960276339		0.969101273	0.97710718
≤ 46	SVM	0.853019958	0.93955095		0.906906151	0.975026015
≤ 46	XGB	0.778098739	0.949913644		0.96181071	0.976066597
≤ 47	GLM	0.78535692	0.946166395		0.909286348	0.9632572
≤ 47	RF	0.923032252	0.95269168		0.95610594	0.974180735
≤ 47	SVM	0.883945323	0.967373573		0.893284574	0.973187686
≤ 47	XGB	0.828977933	0.946166395		0.878025266	0.953326713
≤ 48	GLM	0.878537026	0.956790123		0.893962076	0.957386364
≤ 48	RF	0.923765804	0.962962963		0.95413802	0.96969697
≤ 48	SVM	0.892910897	0.962962963		0.921000591	0.96969697
≤ 48	XGB	0.871086695	0.949074074		0.888842685	0.961174242
≤ 49	GLM	0.842827004	0.944117647		0.884869432	0.956834532
≤ 49	RF	0.932934467	0.952941176		0.930675883	0.981115108
≤ 49	SVM	0.882087289	0.966176471		0.925345622	0.966726619
≤ 49	XGB	0.852304193	0.944117647		0.89984639	0.958633094
≤ 50	GLM	0.837510443	0.947148818		0.896639419	0.949615713
≤ 50	RF	0.797493734	0.940194715		0.88969119	0.951323655
≤ 50	SVM	0.735254804	0.924895688		0.68564941	0.954739539
≤ 50	XGB	0.82428293	0.936022253		0.890735695	0.947907771
≤ 51	GLM	0.872569444	0.92989418		0.892112611	0.944535073
≤ 51	RF	0.833752395	0.92989418		0.876204297	0.946982055
≤ 51	SVM	0.79194205	0.933862434		0.817498593	0.933931485
≤ 51	XGB	0.846791188	0.92989418		0.880028252	0.947797716
≤ 52	GLM	0.825573777	0.93718593		0.863145167	0.93725794
≤ 52	RF	0.82316811	0.929648241		0.850685786	0.938032533
≤ 52	SVM	0.75692525	0.922110553		0.788601413	0.934159566
≤ 52	XGB	0.831541879	0.927135678		0.846869569	0.938032533
≤ 53	GLM	0.827361447	0.935560859		0.853823701	0.938970588
≤ 53	RF	0.853979765	0.928400955		0.845689544	0.938970588
≤ 53	SVM	0.805192945	0.910501193		0.750173841	0.938235294
≤ 53	XGB	0.83196347	0.935560859		0.856815389	0.943382353
≤ 54	GLM	0.843442031	0.928733032		0.882503113	0.938904494
≤ 54	RF	0.831104039	0.926470588		0.853013007	0.931882022

≤ 54	SVM	0.790815307	0.932126697		0.80959596	0.9375
≤ 54	XGB	0.833440954	0.926470588		0.891331119	0.933988764
≤ 55	GLM	0.816898328	0.925646552		0.851307741	0.928475936
≤ 55	RF	0.806476964	0.920258621		0.83166942	0.928475936
≤ 55	SVM	0.756235735	0.90625		0.731061885	0.922459893
≤ 55	XGB	0.78267525	0.920258621		0.866143807	0.928475936
≤ 60	GLM	0.809771296	0.894425676		0.873043704	0.907725322
≤ 60	RF	0.784613149	0.896114865		0.858648586	0.91416309
≤ 60	SVM	0.658112784	0.897804054		0.77747596	0.887339056
≤ 60	XGB	0.791702711	0.893581081		0.87054903	0.906652361
≤ 65	GLM	0.813875668	0.886426593		0.855487803	0.883355765
≤ 65	RF	0.798825435	0.894736842		0.816722707	0.871691341
≤ 65	SVM	0.720966327	0.861495845		0.755686768	0.86855092
≤ 65	XGB	0.811077194	0.885041551		0.856267392	0.885150292
≤ 70	GLM	0.797022895	0.865065752		0.842223092	0.863844394
≤ 70	RF	0.793662985	0.866209262		0.824030843	0.856216629
≤ 70	SVM	0.709253164	0.86049171		0.75825163	0.845156369
≤ 70	XGB	0.807561017	0.865637507		0.847189459	0.8668955
≤ 75	GLM	0.793988392	0.844285714		0.829473082	0.853118712
≤ 75	RF	0.786573265	0.847619048		0.811194257	0.845405768
≤ 75	SVM	0.653210883	0.853333333		0.701599898	0.850771294
≤ 75	XGB	0.813231243	0.851904762		0.837537408	0.851441985
ALL	GLM	0.74815658	0.825197709		0.803035755	0.836578753
ALL	RF	0.742352445	0.819198255		0.786627295	0.839783091
ALL	SVM	0.669266845	0.827379329		0.722417958	0.83386739
ALL	XGB	0.771037867	0.830379056		0.813710721	0.836085778
≤ 80	GLM	0.768905097	0.831844029		0.818175239	0.848903575
≤ 80	RF	0.747318829	0.828594639		0.802342232	0.835386002
≤ 80	SVM	0.724993951	0.829406986		0.765827601	0.842895764
≤ 80	XGB	0.774253114	0.834687246		0.832648711	0.847702013

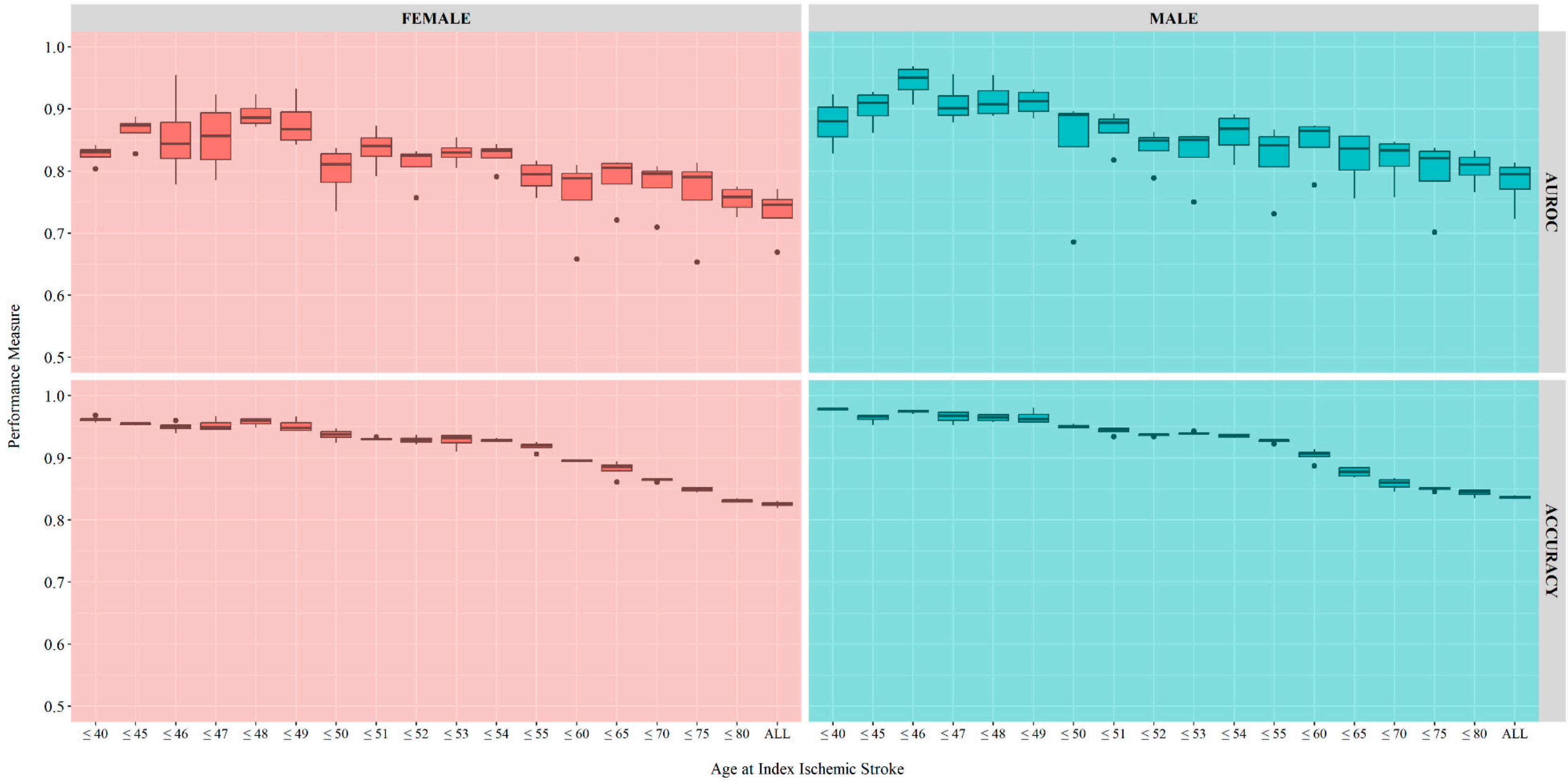

Figure S1. Model performance (AUROC and Accuracy) for different models. Performance measures for all the models are listed in Table S8.
